# Explainable machine learning model for in-hospital hypoglycemia risk in patients with latent autoimmune diabetes in adults

**DOI:** 10.3389/fimmu.2026.1870438

**Published:** 2026-07-27

**Authors:** Qiang Zhang, Xinyi Liu, Yiting Ni, Haojie Zhou, Guniqing Zhu, Ying Wang, Ran Li, Yuan Zhao, Xiangfu Gu, Xiaoyu Cai, Xinmei Nie, Qian Tang, Ruonan Lin, Jianjun Zou, Xiaoli Zhu

**Affiliations:** 1 School of Nursing, Dali University, Yunnan, China; 2 Department of Pharmacy, Nanjing First Hospital, Nanjing Medical University, Jiangsu, China; 3 School of Basic Medicine and Clinical Pharmacy, China Pharmaceutical University, Jiangsu, China; 4 Department of Cardiovascular Medicine, The Second Affiliated Hospital of Chongqing Medical University, Chongqing, China; 5 First Clinical Medical College, Southern Medical University, Guangdong, China; 6 Department of Endocrinology, The First Affiliated Hospital of Dali University, Yunnan, China; 7 School of Public Health, Dali University, Yunnan, China; 8 Patient Service Center, The First Affiliated Hospital of Dali University, Yunnan, China; 9 Department of Critical Care Medicine, The Second Affiliated Hospital of Chongqing Medical University, Chongqing, China

**Keywords:** extreme gradient boosting, hypoglycemia, latent autoimmune diabetes in adults, machine learning, predictive model

## Abstract

**Background:**

Latent autoimmune diabetes in adults (LADA) is characterized by progressive β-cell impairment and severe glycemic lability, predisposing patients to in-hospital hypoglycemia. Few tailored risk-stratification models exist for this population. This study aimed to develop and validate an interpretable machine learning model using routine clinical data to predict in-hospital hypoglycemia in LADA inpatients.

**Methods:**

This multicenter retrospective study recruited participants from five Chinese tertiary hospitals between January 2019 and September 2025. Data from four centers formed the derivation cohort, and the remaining center served as the independent external validation cohort. The primary endpoint was in-hospital hypoglycemia (blood glucose < 3.9 mmol/L). Three machine learning models, including logistic regression, random forest, and XGBoost, were developed using routine clinical data and assessed for discrimination, calibration, and clinical utility. SHAP analysis was applied to improve model interpretability. Exploratory subgroup analyses in the internal validation cohort examined model performance across clinical subgroups.

**Results:**

A total of 752 LADA inpatients were enrolled. The incidence of in-hospital hypoglycemia was 44.8% in the derivation cohort and 54.4% in the external validation cohort. Six core predictive factors were identified: largest amplitude of glycemic excursion, fasting C-peptide, glycated hemoglobin, sex, insulin pump use, and previous hypoglycemia. The three models yielded numerically variable discriminative performance across cohorts. Pairwise DeLong tests indicated no statistically significant differences in the AUROC among the three algorithms during external validation. All models showed comparable calibration and threshold-dependent predictive performance in the external cohort. XGBoost was selected as the final model after comprehensive evaluation. Fasting C-peptide was identified as the most influential predictor. Exploratory subgroup analyses demonstrated generally stable model performance across clinical strata. These findings are limited by small subgroup sample sizes and wide confidence intervals, and thus cannot be generalized to external populations. Sensitivity analysis suggested that model performance was not predominantly dependent on the retained glucose-derived predictor.

**Conclusions:**

The interpretable XGBoost model showed acceptable discrimination, calibration, and potential clinical utility for in-hospital hypoglycemia risk stratification in patients with LADA. This pragmatic predictive tool has the potential to support individualized inpatient glycemic management and facilitate targeted clinical intervention for LADA populations.

## 1 Introduction

Latent autoimmune diabetes in adults (LADA) is a distinct subtype of diabetes mellitus sharing features with type 1 diabetes mellitus (T1DM) and type 2 diabetes mellitus (T2DM) (1), and is characterized by progressive autoimmune-mediated pancreatic β-cell failure (2). Its early clinical manifestations mimic those of T2DM, which often leads to underdiagnosis or misdiagnosis (3). While LADA patients preserve partial β-cell function at diagnosis and do not need immediate insulin treatment (4, 5), pancreatic β-cell function deteriorates continuously over time (6). Most patients eventually require long-term insulin therapy to maintain adequate glycemic control (7).

The progressive loss of β-cell function causes unstable glycemic control in LADA patients and further elevates their risk of hypoglycemia (8). As one of the most common acute complications of diabetes (9), hypoglycemia may cause palpitations, diaphoresis, and impaired consciousness (10). In severe cases, it progresses to coma, permanent cerebral damage, or even death (11). Unstable glycemic regulation, combined with hospitalization-related factors including dietary changes, physical stress, and insulin dose adjustment, substantially raises the risk of in-hospital hypoglycemia among hospitalized LADA patients (12).

Numerous studies have developed predictive models for hypoglycemia based on clinical indicators in diabetic populations (13). However, most of these models were developed for patients with T1DM, T2DM or mixed diabetic cohorts, rather than specifically for LADA patients (14). Given the unique pathophysiology of LADA, including autoimmune islet destruction and progressive β-cell exhaustion (15), simply applying existing models to LADA may result in predictive bias and inadequate hypoglycemia risk stratification in this population.

Machine learning is well-suited to capture complex relationships and facilitate risk stratification in diabetes research (16), while interpretable models are still rarely developed for LADA. Furthermore, some previous studies on LADA screening or risk stratification have relied on genetic testing, comprehensive islet autoantibody assays, or specialized biomarkers that are not widely available in routine clinical practice (17, 18).

Therefore, this study employed a multicenter cohort of hospitalized LADA patients and routinely available clinical variables to construct and validate an interpretable machine learning model for predicting in-hospital hypoglycemia risk.

## 2 Materials and methods

### 2.1 Study design and population

This multicenter retrospective cohort study analyzed inpatient electronic health records from five tertiary hospitals across China from January 2019 to September 2025. Patients with a confirmed diagnosis of LADA, were enrolled in accordance with prespecified inclusion and exclusion criteria. Participants from four hospitals were assigned to the derivation cohort for model development, while patients from the remaining hospital formed an independent external validation cohort.

The inclusion criteria were as follows: (1) age ≥ 18 years; (2) clinically diagnosed with LADA in accordance with standard diagnostic criteria; (3) complete inpatient blood glucose monitoring data available for outcome assessment.

The exclusion criteria were as follows: (1) diagnosed with other types of diabetes, including classic T1DM, T2DM or other specific diabetes subtypes; (2) admitted with critical conditions such as severe infection, vital organ failure or hematological diseases that could substantially interfere with glycemic control; (3) missing key blood glucose indicators or laboratory test results; (4) incomplete clinical records; (5) hospital stay shorter than 24 hours.

This study was approved by the Institutional Ethics Committee. All raw data were fully anonymized prior to analysis to protect patient confidentiality. Given the retrospective nature of the study, the requirement for written informed consent was waived by the ethics committee. The study design and reporting complied with the TRIPOD + AI Statement (19).

### 2.2 Study time frame and in-hospital variable window

All analyses were restricted to the inpatient period. An individualized reference time point was defined for each patient to distinguish predictor assessment from outcome ascertainment and reduce outcome leakage. For patients with in-hospital hypoglycemia, this time point referred to the last non-hypoglycemic glucose measurement prior to the first hypoglycemic episode. For those without hypoglycemia, one non-hypoglycemic inpatient glucose measurement was randomly selected via a prespecified sampling protocol as the reference time point.

**Figure 1:**
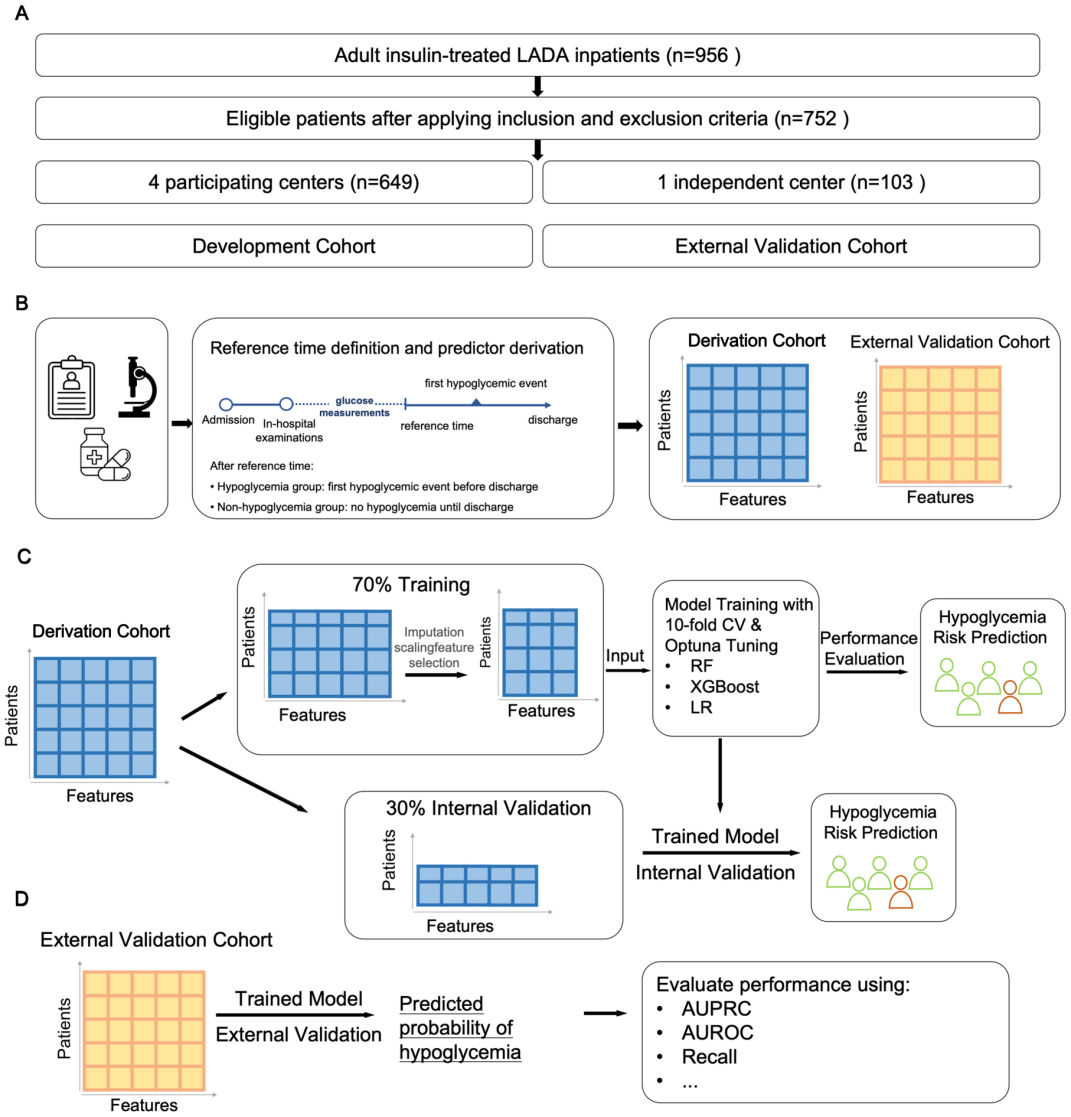
Study design and analytical workflow. **(A)** Flowchart of patient enrollment and cohort construction. **(B)** Definition of the reference time point and predictor window. **(C, D)** Model development, internal validation, and external validation workflow.

All candidate predictors, including demographic characteristics, laboratory parameters, treatment information and glycemic metrics, were extracted from the 24-hour window prior to the reference time point. This unified 24-hour period was applied to all participants to ensure consistent derivation of glycemic variables. In-hospital hypoglycemia was identified throughout the interval from the reference time point to discharge. Notably, blood glucose measurements used to define hypoglycemic events were not included in the calculation of predictive variables. The overall study framework and time window definitions are illustrated in **Figure 1**.

### 2.3 Candidate variables and outcome

Candidate predictors were selected based on a comprehensive literature review and Delphi expert consensus. All variables were directly extracted from electronic health records without additional examinations. A total of 66 variables were collected prior to feature selection, including demographic characteristics, diabetes duration, medical history, treatment regimens, biochemical indicators, and dynamic glycemic parameters derived from continuous inpatient glucose monitoring. Detailed information on all variables, operational definitions, measurement time points and pre-imputation missing rates is presented in **Supplementary Table 1**.

The primary outcome was in-hospital hypoglycemia, treated as a binary endpoint. Hypoglycemia was defined according to the three-tier classification criteria recommended by the American Diabetes Association (20): level 1 (3.0 mmol/L ≤ blood glucose < 3.9 mmol/L), level 2 (blood glucose < 3.0 mmol/L), and level 3 (severe hypoglycemia accompanied by altered consciousness requiring external assistance). Any episode meeting the criteria for level 1 or above was regarded as a hypoglycemic event.

### 2.4 Data preprocessing and statistical analysis

All statistical analyses were conducted using SPSS 25.0 and Python 3.10.4. The main software and package versions applied for model development and interpretation are listed in **Supplementary Table 2**. A two-sided P value < 0.05 was considered statistically significant. Continuous variables were expressed as mean ± standard deviation (SD) for normally distributed data and median (interquartile range, IQR) for non-normal data, with nonparametric tests adopted for between-group comparisons. Categorical variables were summarized as counts and percentages.

Standardized data preprocessing was performed prior to model construction. Erroneous entries, non-standard formats, and invalid values were removed. Outliers were processed via winsorization (21). Laboratory indicators were interpreted according to clinical reference ranges. For indicators without unified cutoffs, the interquartile range method and 3-standard-deviation rule were adopted.

The derivation cohort was randomly divided into a training set and an internal validation set at a 7:3 ratio. The training set was used for feature selection, model training and hyperparameter optimization, whereas the internal validation set was applied to preliminarily assess model reliability and stability. Variables with a missing rate exceeding 40% in the derivation cohort were excluded in advance. This cutoff was adopted per common practice in clinical prediction modeling to remove variables with excessive missing data while retaining clinically meaningful candidates for subsequent screening (22).

Remaining missing values were handled using multiple imputation by chained equations (MICE) in the primary analysis. To prevent data leakage, imputation rules and preprocessing pipelines, including standardization and one-hot encoding, were established solely on the training set and then applied consistently to the internal and external validation cohorts (23). Standardized mean differences (SMDs) were calculated to compare variable distributions before and after imputation across the derivation and external validation cohorts, as shown in **Supplementary Tables 3**, **4**. Continuous variables were standardized using the z-score approach, and categorical variables were converted via one-hot encoding (24).

This study complied with the widely accepted guideline that each predictor should correspond to no fewer than 10 outcome events (25). Since the final model was designed to contain 5–10 predictors, the dataset contained at least 50 outcome events to guarantee robust model estimation.

### 2.5 Model development, feature selection and validation

A multistep feature selection strategy was implemented in the training set. First, least absolute shrinkage and selection operator (LASSO) logistic regression with 10-fold cross-validation was applied to screen candidate predictors, aiming to generate a concise and stable variable set and mitigate overfitting. Variables with non-zero regression coefficients under the optimal penalty parameter were retained (26).

Variance inflation factor (VIF) was then calculated to detect multicollinearity among the selected variables. Variables with a VIF greater than 5 were excluded, as this threshold is widely used to identify severe multicollinearity (27). The remaining variables were further evaluated for cross-center generalizability to determine the final predictors for model construction.

Three predictive models, including logistic regression, random forest and extreme gradient boosting (XGBoost), were developed in parallel. Hyperparameter optimization was performed using the Optuna framework integrated with the tree-structured Parzen estimator algorithm under 10-fold stratified cross-validation (28). Class weights were applied during training to address class imbalance.

Model performance was evaluated in both internal and external validation cohorts. The area under the receiver operating characteristic curve (AUROC) was defined as the primary outcome metric. Additional evaluation indicators included the area under the precision-recall curve (AUPRC), calibration curves, Brier score and decision curve analysis (DCA). Bootstrap resampling with 1000 iterations was performed to calculate 95% confidence intervals for AUROC and AUPRC.

Pairwise comparisons of AUROC across the three models were conducted via the DeLong tests, using paired predicted probabilities from the same validation cohort (29). The AUROC difference was calculated as the value of model 1 minus that of model 2. All statistical tests were two-tailed, and P < 0.05 indicated statistically significant. The final model was selected by comprehensively considering discriminative ability, calibration performance, net clinical benefit derived from DCA, and threshold-based classification performance.

The optimal classification threshold for each model was determined in the training set by maximizing the Youden index. These cutoffs were applied uniformly to internal and external validation cohorts without further adjustment (30). SHapley Additive exPlanations (SHAP) was adopted to interpret the final model (31). Subgroup analyses were also conducted within clinically relevant strata.

A series of sensitivity analyses were performed to evaluate model robustness. First, the final model was retrained after excluding each predictor individually (32). Second, several alternative missing data approaches, including median imputation, missing-indicator method, complete-case analysis and K-nearest neighbor (KNN) imputation, were tested to assess whether model performance depended on the primary MICE strategy (33). Third, analyses were restricted to participants with complete islet autoantibody data. The original model was compared with an expanded version incorporating GAD, IAA, ZnT8, IA-2 and ICA to explore the incremental predictive value of autoantibody markers (34).

## 3 Results

### 3.1 Study population and feature selection

**Figure 2:**
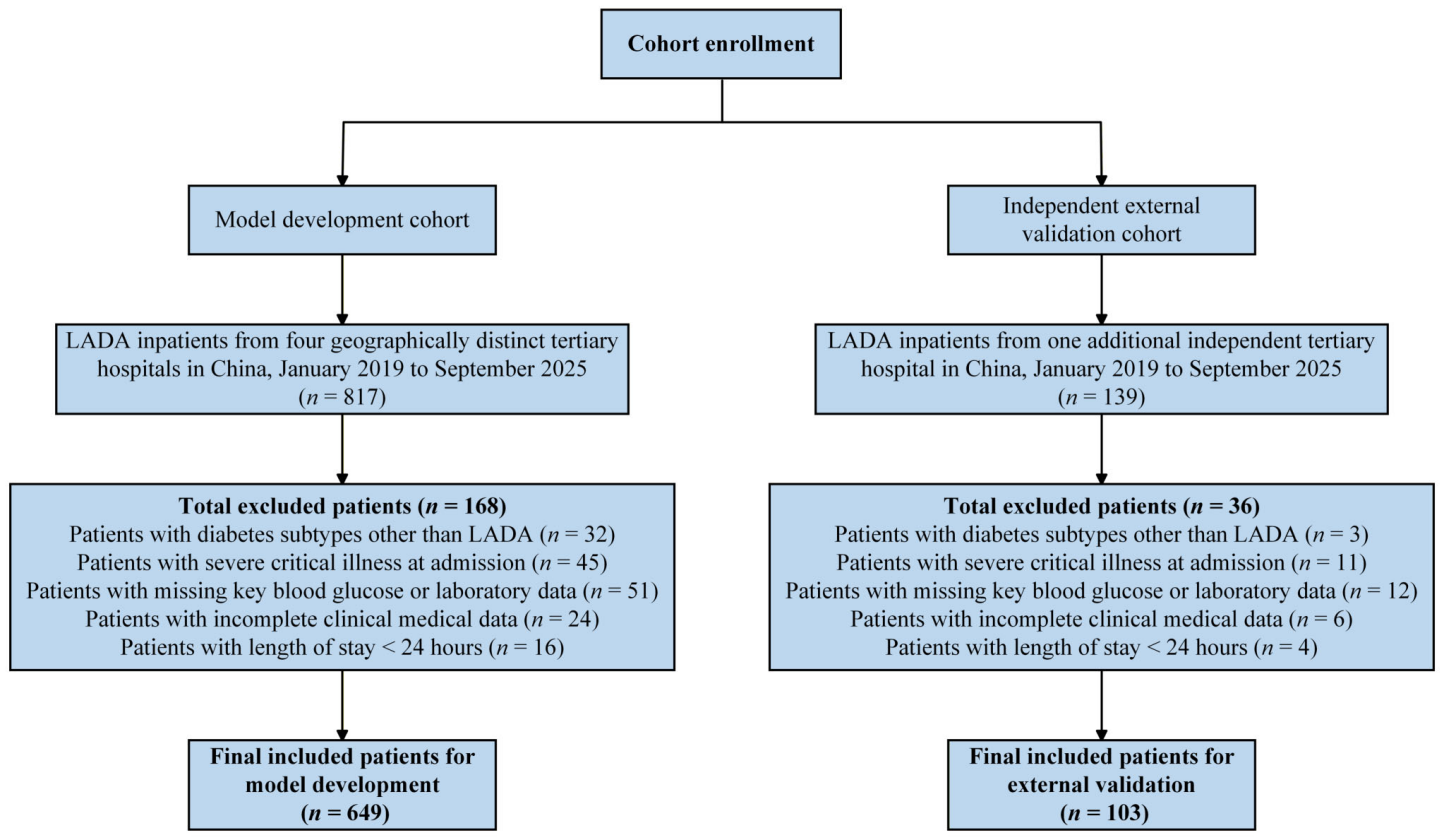
Flowchart of participant enrollment and exclusion.

A total of 956 medical records were initially screened, and 752 eligible participants were finally enrolled. The derivation cohort consisted of 649 patients from four medical centers, which was randomly divided into a training set (*n* = 455) and an internal validation set (*n* = 194). The remaining 103 patients from the fifth center formed the independent external validation cohort. The detailed flow of participant enrollment and exclusion is presented in **Figure 2**.

In-hospital hypoglycemia occurred in 44.8% (291/649) of the derivation cohort and 54.4% (56/103) of the external validation cohort. Baseline characteristics were generally balanced between the training and internal validation sets, with ethnicity being the only significant difference (**Table 1**). Baseline differences between the overall derivation cohort and external validation cohort are summarized in **Table 2**.

**Table 1:** Baseline characteristics of the training and internal validation cohorts.

Variable		Training set (*n* = 455)	Internal validation set (*n* = 194)	*P* value	SMD
Sex (*n*, %)	Male	287 (63.1%)	113 (58.2%)	0.253	0.10
	Female	168 (36.9%)	81 (41.8%)		
Age [M (P_25_, P_75_), year]		50.0 (40.0, 60.0)	50.5 (37.2, 61.0)	0.845	0.03
Marital status (*n*, %)	Married	389 (85.5%)	161 (83.0%)	0.788	0.07
	Unmarried	39 (8.6%)	20 (10.3%)		
	Divorced	19 (4.2%)	8 (4.1%)		
	Widowed	8 (1.8%)	5 (2.6%)		
Ethnicity (*n*, %)	Han	307 (67.5%)	145 (74.7%)	0.230	0.16
	Bai	86 (18.9%)	30 (15.5%)		
	Yi	31 (6.8%)	12 (6.2%)		
	Other minorities	31 (6.8%)	7 (3.6%)		
Education level (*n*, %)	Illiterate/Primary school education	123 (27.0%)	52 (26.8%)	0.601	0.08
	Junior/Senior high school	229 (50.3%)	91 (46.9%)		
	University and above	103 (22.6%)	51 (26.3%)		
Insurance status (*n*, %)	UEBMI	191 (42.0%)	82 (42.3%)	0.677	0.09
	URRBMI	248 (54.5%)	108 (55.7%)		
	Self-payment	16 (3.5%)	4 (2.1%)		
BMI [M (P_25_, P_75_), kg/m^2^]		21.4 (19.2, 23.4)	21.4 (19.8, 23.6)	0.376	0.05
Smoking status (*n*, %)	Yes	231 (50.8%)	81 (41.8%)	0.039	0.18
	No	224 (49.2%)	113 (58.2%)		
Duration of smoking [M (P_25_, P_75_), year]		25.6 (17.5, 32.5)	27.8 (19.1, 35.2)	0.158	0.12
Alcohol use (*n*, %)	Yes	136 (29.9%)	40 (20.6%)	0.016	0.21
	No	319 (70.1%)	151 (79.4%)		
Duration of alcohol use [M (P_25_, P_75_), year]		22.0 (15.4, 30.0)	21.0 (15.1, 30.6)	0.894	0.01
Age at diabetes onset [M (P_25_, P_75_), year]		44.0 (35.0, 54.0)	46.0 (33.0, 54.0)	0.668	0.07
Diabetes duration [M (P_25_, P_75_), year]		3.0 (0.0, 8.0)	3.0 (0.0, 8.8)	0.286	0.09
Previous hypoglycemia (*n*, %)	Yes	71 (15.6%)	40 (20.6%)	0.139	0.13
	No	384 (84.4%)	151 (79.4%)		
Family history of diabetes (*n*, %)	Yes	81 (17.8%)	32 (16.5%)	0.735	0.03
	No	374 (82.2%)	162 (83.5%)		
History of hypertension (*n*, %)	Yes	87 (19.1%)	44 (22.7%)	0.336	0.09
	No	368 (80.9%)	150 (77.3%)		
Ketosis (*n*, %)	Yes	172 (37.8%)	75 (38.7%)	0.860	0.02
	No	283 (62.2%)	119 (61.3%)		
Diabetic ketoacidosis (*n*, %)	Yes	57 (12.5%)	30 (15.5%)	0.316	0.08
	No	398 (87.5%)	164 (84.5%)		
Diabetic retinopathy (*n*, %)	Yes	80 (17.6%)	36 (18.6%)	0.823	0.03
	No	375 (82.4%)	158 (81.4%)		
Diabetic kidney disease (*n*, %)	Yes	50 (11.0%)	17 (8.8%)	0.481	0.07
	No	405 (89.0%)	177 (91.2%)		
Diabetic neuropathy (*n*, %)	Yes	325 (71.4%)	137 (70.6%)	0.850	0.02
	No	130 (28.6%)	57 (29.4%)		
Diabetic vascular disease (*n*, %)	Yes	130 (28.6%)	51 (26.3%)	0.568	0.05
	No	325 (71.4%)	143 (73.7%)		
Diabetic foot (*n*, %)	Yes	6 (1.3%)	0 (0.0%)	0.186	0.16
	No	449 (98.7%)	194 (100%)		
Number of diabetic complications [M (P_25_, P_75_)]		2.0 (1.0, 2.0)	2.0 (1.0, 2.0)	0.467	0.07
Sleep disorder (*n*, %)	Yes	11 (2.4%)	8 (4.1%)	0.307	0.10
	No	444 (97.6%)	186 (95.9%)		
Polypharmacy (*n*, %)	Yes	385 (84.6%)	160 (82.5%)	0.486	0.06
	No	70 (15.4%)	34 (17.5%)		
Oral hypoglycemic agent use (*n*, %)	Yes	244 (53.6%)	99 (51.0%)	0.549	0.05
	No	211 (46.4%)	95 (49.0%)		
Insulin regimen (*n*, %)	CSII	42 (9.2%)	23 (11.9%)	0.313	0.12
	MDI	335 (73.6%)	146 (75.3%)		
	LFIR	78 (17.1%)	25 (12.9%)		
Insulin pump use (*n*, %)	Yes	46 (10.1%)	23 (11.9%)	0.265	0.10
	No	399 (89.9%)	171 (88.1%)		
Total daily insulin dose [M (P_25_, P_75_), U]		31.0 (23.0, 40.0)	31.0 (24.0, 40.8)	0.965	0.01
Premeal insulin use [M (P_25_, P_75_), U]		19.0 (14.0, 26.0)	19.7 (14.0, 26.0)	0.870	0.01
Basal insulin use [M (P_25_, P_75_), U]		12.0 (8.0, 16.0)	12.0 (8.0, 17.0)	0.908	0.03
Insulin type (*n*, %)	1	122 (26.8%)	53 (27.3%)	0.312	0.11
	2	322 (70.8%)	138 (71.1%)		
	3	11 (2.4%)	2 (1.0%)		
	4	0 (0.0%)	1 (0.5%)		
PPGE [M (P_25_, P_75_), mmol/L]		0.7 (-0.9, 2.1)	0.7 (-0.8, 2.3)	0.770	0.06
LAGE [M (P_25_, P_75_), mmol/L]		7.2 (4.8, 10.1)	6.7 (4.3, 10.3)	0.313	0.05
Mean blood glucose [M (P_25_, P_75_), mmol/L]		9.5 (8.0, 11.2)	9.5 (8.0, 11.1)	0.973	0.02
HbA1c [M (P_25_, P_75_), %]		10.6 (9.0, 13.3)	10.4 (8.5, 12.6)	0.201	0.08
Fasting C-peptide [M (P_25_, P_75_), ng/mL]		0.4 (0.1, 1.0)	0.5 (0.1, 1.0)	0.693	0.01
0.5-h C-peptide [M (P_25_, P_75_), ng/mL]		0.5 (0.2, 1.2)	0.5 (0.2, 1.2)	0.870	0.03
1-h C-peptide [M (P_25_, P_75_), ng/mL]		0.7 (0.3, 1.6)	0.7 (0.3, 1.7)	0.806	0.01
2-h C-peptide [M (P_25_, P_75_), ng/mL]		1.0 (0.4, 2.5)	1.1 (0.4, 2.6)	0.491	0.09
Serum creatinine [M (P_25_, P_75_), μmol/L]		62.0 (52.0, 75.2)	61.7 (52.6, 74.9)	0.987	0.05
eGFR [M (P_25_, P_75_), mL/(min·1.73m²)]		106.2 (93.6, 117.5)	104.1 (91.1, 117.0)	0.404	0.08
Total protein [M (P_25_, P_75_), g/L]		66.2 (59.7, 71.2)	65.5 (59.7, 70.5)	0.393	0.08
Total cholesterol [M (P_25_, P_75_), mmol/L]		4.4 (3.8, 5.2)	4.5 (3.8, 5.4)	0.619	0.05
Triglycerides [M (P_25_, P_75_), mmol/L]		1.2 (0.8, 1.8)	1.1 (0.8, 1.6)	0.164	0.09
LDL-C [M (P_25_, P_75_), mmol/L]		2.4 (1.9, 3.0)	2.5 (1.9, 3.2)	0.367	0.10
HDL-C [M (P_25_, P_75_), mmol/L]		1.2 (1.0, 1.5)	1.3 (1.0, 1.6)	0.553	0.06
Hemoglobin [M (P_25_, P_75_), g/L]		140.0 (127.0, 154.0)	139.0 (127.0, 151.0)	0.373	0.09
RBC count [M (P_25_, P_75_), ×10¹²/L]		4.7 (4.3, 5.1)	4.6 (4.2, 5.0)	0.222	0.08
WBC count [M (P_25_, P_75_), ×10^9^/L]		6.1 (5.0, 7.5)	6.3 (5.2, 7.9)	0.086	0.11
CRP [M (P_25_, P_75_), mg/L]		3.2 (2.0, 5.4)	3.1 (1.6, 5.6)	0.730	0.04
25-hydroxyvitamin D [M (P_25_, P_75_), ng/mL]		28.0 (19.2, 39.9)	29.6 (19.8, 40.1)	0.284	0.13
Urinary microalbumin [M (P_25_, P_75_), mg/L]		15.2 (9.9, 30.3)	15.3 (9.8, 30.2)	0.860	0.07
Urinary protein [M (P_25_, P_75_)]		0.0 (0.0, 0.2)	0.0 (0.0, 0.2)	0.684	0.02
GAD antibody (*n*, %)	Positive	315 (69.2%)	144 (74.2%)	0.221	0.11
	Negative	140 (30.8%)	50 (25.8%)		
ZnT8 antibody (*n*, %)	Positive	55 (12.1%)	23 (11.9%)	1.000	0.01
	Negative	400 (87.9%)	171 (88.1%)		
IAA (*n*, %)	Positive	71 (15.6%)	34 (17.5%)	0.561	0.05
	Negative	384 (84.4%)	160 (82.5%)		
IA-2 antibody (*n*, %)	Positive	42 (9.2%)	14 (7.2%)	0.448	0.07
	Negative	413 (90.8%)	180 (92.8%)		
ICA (*n*, %)	Positive	45 (9.9%)	16 (8.2%)	0.536	0.10
	Negative	410 (90.1%)	178 (91.8%)		
Serum potassium [M (P_25_, P_75_), mmol/L]		4.0 (3.8, 4.3)	4.0 (3.8, 4.2)	0.910	0.05
Serum sodium [M (P_25_, P_75_), mmol/L]		139.0 (136.1, 141.0)	138.8 (136.0, 141.0)	0.542	0.05
Serum calcium [M (P_25_, P_75_), mmol/L]		2.2 (2.2, 2.4)	2.2 (2.2, 2.4)	0.744	0.05
In-hospital hypoglycemia occurrence (*n*, %)	Yes	204 (44.8%)	87 (44.8%)	1.000	0.00
	No	251 (55.2%)	107 (55.2%)		
Length of stay [M (P_25_, P_75_), day]		9.0 (7.0, 12.0)	9.0 (7.0, 12.0)	0.504	0.10

UEBMI, Urban Employee Basic Medical Insurance; URRBMI, Urban, Rural Resident Basic Medical Insurance; CSII, Continuous Subcutaneous Insulin Infusion; MDI, Multiple Daily Injections; LFIR, Low Frequency Insulin Regimen; PPGE, Postprandial Plasma Glucose Excursion; LAGE, Largest Amplitude of Glycemic Excursion; BMI, Body Mass Index; HbA1c, Glycated Hemoglobin A1c; C-peptide, Connecting peptide; eGFR, estimated Glomerular Filtration Rate; TC, Total Cholesterol; TG, Triglycerides; LDL-C, Low-Density Lipoprotein Cholesterol; HDL-C, High-Density Lipoprotein Cholesterol; RBC, Red Blood Cell; WBC, White Blood Cell; CRP, C-Reactive Protein; 25(OH)D, 25-hydroxyvitamin D; GAD, Glutamic Acid Decarboxylase antibody; ZnT8, Zinc Transporter 8 antibody; IAA, Insulin Autoantibody; IA-2, Insulinoma-Associated Antigen 2 antibody; ICA, Islet Cell Antibody; SMD, Standardized Mean Difference; Cr, Creatinine; U, Insulin unit; NA, Not Applicable.

**Table 2:** Baseline characteristics of the derivation and external validation cohorts.

Variable		Derivation cohort (*n* = 649)	External validation cohort (*n* = 103)	*P* value	SMD
Sex (*n*, %)	Male	400 (61.6%)	54 (52.4%)	0.083	0.19
	Female	249 (38.4%)	49 (47.6%)		
Age [M (P_25_, P_75_), year]		50.0 (40.0, 60.0)	50.0 (33.5, 58.0)	0.095	0.18
Marital status (*n*, %)	Married	550 (84.7%)	75 (72.8%)	< 0.001	0.47
	Unmarried	59 (9.1%)	6 (5.8%)		
	Divorced	27 (4.2%)	20 (19.4%)		
	Widowed	13 (2.0%)	2 (1.9%)		
Ethnicity (*n*, %)	Han	452 (69.6%)	87 (84.5%)	0.019	0.35
	Bai	116 (17.9%)	10 (9.7%)		
	Yi	43 (6.6%)	4 (3.9%)		
	Other minorities	38 (5.9%)	2 (1.9%)		
Education level (*n*, %)	Illiterate/Primary school education	175 (27.0%)	41 (39.8%)	0.026	0.27
	Junior/Senior high school	320 (49.3%)	44 (42.7%)		
	University and above	154 (23.7%)	18 (17.5%)		
Insurance status (*n*, %)	UEBMI	273 (42.1%)	39 (37.9%)	0.696	0.09
	URRBMI	356 (54.9%)	61 (59.2%)		
	Self-payment	20 (3.1%)	3 (2.9%)		
BMI [M (P_25_, P_75_), kg/m^2^]		21.4 (19.4, 23.5)	20.8 (19.5, 22.7)	0.201	0.15
Smoking status (*n*, %)	Yes	312 (48.1%)	44 (42.7%)	0.340	0.11
	No	337 (51.9%)	59 (57.3%)		
Duration of smoking [M (P_25_, P_75_), year]		26.0 (18.0, 34.0)	10.0 (0.0, 20.0)	< 0.001	1.12
Alcohol use(*n*, %)	Yes	176 (27.1%)	15 (14.6%)	0.007	0.31
	No	473 (72.9%)	88 (85.4%)		
Duration of alcohol use [M (P_25_, P_75_), year]		21.6 (15.2, 30.0)	1.5 (0.0, 7.9)	< 0.001	1.90
Age at diabetes onset [M (P_25_, P_75_), year]		45.0 (35.0, 54.0)	47.0 (36.0, 57.5)	0.188	0.13
Diabetes duration [M (P_25_, P_75_), year]		3.0 (0.0, 8.0)	6.9 (1.6, 9.0)	< 0.001	0.26
Previous hypoglycemia (*n*, %)	Yes	111 (17.1%)	36 (35.0%)	< 0.001	0.41
	No	538 (82.9%)	67 (65.0%)		
Family history of diabetes (*n*, %)	Yes	113 (17.4%)	13 (12.6%)	0.258	0.13
	No	536 (82.6%)	90 (87.4%)		
History of hypertension (*n*, %)	Yes	131 (20.2%)	15 (14.6%)	0.227	0.15
	No	518 (79.8%)	88 (85.4%)		
Ketosis (*n*, %)	Yes	247 (38.1%)	50 (48.5%)	0.051	0.21
	No	402 (61.9%)	53 (51.5%)		
Diabetic ketoacidosis (*n*, %)	Yes	87 (13.4%)	16 (15.5%)	0.539	0.06
	No	562 (86.6%)	87 (84.5%)		
Diabetic retinopathy (*n*, %)	Yes	116 (17.9%)	3 (2.9%)	< 0.001	0.49
	No	533 (82.1%)	100 (97.1%)		
Diabetic kidney disease (*n*, %)	Yes	67 (10.3%)	8 (7.8%)	0.484	0.09
	No	582 (89.7%)	95 (92.2%)		
Diabetic neuropathy (*n*, %)	Yes	462 (71.2%)	65 (63.1%)	0.105	0.17
	No	187 (28.8%)	38 (36.9%)		
Diabetic vascular disease (*n*, %)	Yes	181 (27.9%)	9 (8.7%)	< 0.001	0.50
	No	468 (72.1%)	94 (91.3%)		
Diabetic foot (*n*, %)	Yes	6 (0.9%)	1 (1.0%)	1.000	0.00
	No	643 (99.1%)	102 (99.0%)		
Number of diabetic complications [M (P_25_, P_75_)]		2.0 (1.0, 2.0)	1.0 (1.0, 2.0)	0.006	0.33
Sleep disorder (*n*, %)	Yes	19 (2.9%)	1 (1.0%)	0.504	0.14
	No	630 (97.1%)	102 (99.0%)		
Polypharmacy (*n*, %)	Yes	545 (84.0%)	73 (70.9%)	0.002	0.31
	No	104 (16.0%)	30 (29.1%)		
Oral hypoglycemic agent use (*n*, %)	Yes	343 (52.9%)	34 (33.0%)	< 0.001	0.40
	No	306 (47.1%)	69 (67.0%)		
Insulin regimen (*n*, %)	CSII	65 (10.0%)	17 (16.5%)	0.138	0.19
	MDI	481 (74.1%)	69 (67.0%)		
	LFIR	103 (15.9%)	17 (16.5%)		
Insulin pump use (*n*, %)	Yes	62 (9.6%)	8 (7.8%)	0.773	0.08
	No	587 (90.4%)	95 (92.2%)		
Total daily insulin dose [M (P_25_, P_75_), U]		31.0 (23.4, 40.0)	30.4 (23.6, 36.0)	0.215	0.18
Premeal insulin use [M (P_25_, P_75_), U]		19.0 (14.0, 26.0)	18.0 (12.0, 23.5)	0.035	0.24
Basal insulin use [M (P_25_, P_75_), U]		12.0 (8.0, 16.8)	13.0 (9.5, 16.0)	0.502	0.00
Insulin type (*n*, %)	1	175 (27.0%)	35 (34.0%)	0.457	0.15
	2	460 (70.9%)	66 (64.1%)		
	3	13 (2.0%)	2 (1.9%)		
	4	1 (0.2%)	0 (0.0%)		
PPGE [M (P_25_, P_75_), mmol/L]		0.7 (-0.8, 2.1)	1.8 (0.6, 3.3)	< 0.001	0.43
LAGE [M (P_25_, P_75_), mmol/L]		7.1 (4.7, 10.2)	7.5 (5.6, 11.6)	0.168	0.16
Mean blood glucose [M (P_25_, P_75_), mmol/L]		9.5 (8.0, 11.2)	9.4 (8.2, 11.6)	0.633	0.08
HbA1c [M (P_25_, P_75_), %]		10.5 (8.9, 13.0)	9.7 (8.5, 11.4)	0.016	0.08
Fasting C-peptide [M (P_25_, P_75_), ng/mL]		0.4 (0.1, 1.0)	0.0 (0.0, 0.4)	< 0.001	0.41
0.5-h C-peptide [M (P_25_, P_75_), ng/mL]		0.5 (0.2, 1.3)	0.1 (0.0, 0.6)	< 0.001	0.58
1-h C-peptide [M (P_25_, P_75_), ng/mL]		0.7 (0.3, 1.6)	0.1 (0.0, 0.7)	< 0.001	0.54
2-h C-peptide [M (P_25_, P_75_), ng/mL]		1.1 (0.4, 2.5)	0.1 (0.0, 1.0)	< 0.001	0.39
Serum creatinine [M (P_25_, P_75_), μmol/L]		62.0 (52.0, 75.0)	57.1 (48.3, 69.9)	0.048	0.07
eGFR [M (P_25_, P_75_), mL/(min·1.73m²)]		105.7 (92.9, 117.4)	106.4 (95.8, 123.5)	0.079	0.26
Total protein [M (P_25_, P_75_), g/L]		65.9 (59.7, 70.8)	70.1 (64.8, 74.3)	< 0.001	0.52
Total cholesterol [M (P_25_, P_75_), mmol/L]		4.4 (3.8, 5.2)	4.3 (3.8, 5.1)	0.661	0.07
Triglycerides [M (P_25_, P_75_), mmol/L]		1.1 (0.8, 1.8)	1.1 (0.8, 1.4)	0.058	0.01
LDL-C [M (P_25_, P_75_), mmol/L]		2.5 (1.9, 3.0)	2.4 (2.0, 3.0)	0.800	0.07
HDL-C [M (P_25_, P_75_), mmol/L]		1.3 (1.0, 1.6)	1.4 (1.2, 1.6)	0.020	0.11
Hemoglobin [M (P_25_, P_75_), g/L]		139.0 (127.0, 153.0)	139.0 (126.2, 152.1)	0.909	0.06
RBC count [M (P_25_, P_75_), ×10¹²/L]		4.7 (4.3, 5.1)	4.8 (4.5, 5.6)	< 0.001	0.24
WBC count [M (P_25_, P_75_), ×10^9^/L]		6.2 (5.0, 7.6)	6.8 (5.0, 8.5)	0.033	0.17
CRP [M (P_25_, P_75_), mg/L]		3.2 (1.8, 5.4)	2.8 (0.8, 4.6)	0.003	0.01
25-hydroxyvitamin D [M (P_25_, P_75_), ng/mL]		28.6 (19.2, 39.9)	19.0 (14.2, 22.8)	< 0.001	0.90
Urinary microalbumin [M (P_25_, P_75_), mg/L]		15.2 (9.9, 30.3)	11.8 (11.0, 32.0)	0.439	0.12
Urinary protein [M (P_25_, P_75_)]		0.0 (0.0, 0.2)	0.0 (0.0, 0.0)	0.006	0.19
GAD antibody (*n*, %)	Positive	459 (70.7%)	65 (63.1%)	0.133	0.16
	Negative	190 (29.3%)	38 (36.9%)		
ZnT8 antibody (*n*, %)	Positive	78 (12.0%)	32 (31.1%)	< 0.001	0.46
	Negative	571 (88.0%)	71 (68.9%)		
IAA (*n*, %)	Positive	105 (16.2%)	26 (25.2%)	0.035	0.22
	Negative	544 (83.8%)	77 (74.8%)		
IA-2 antibody (*n*, %)	Positive	56 (8.6%)	14 (13.6%)	0.141	0.16
	Negative	593 (91.4%)	89 (86.4%)		
ICA (*n*, %)	Positive	61 (9.4%)	30 (29.1%)	< 0.001	0.52
	Negative	588 (90.6%)	73 (70.9%)		
Serum potassium [M (P_25_, P_75_), mmol/L]		4.0 (3.8, 4.3)	4.1 (3.9, 4.3)	0.054	0.10
Serum sodium [M (P_25_, P_75_), mmol/L]		139.0 (136.0, 141.0)	139.0 (137.1, 140.0)	0.800	0.10
Serum calcium [M (P_25_, P_75_), mmol/L]		2.2 (2.2, 2.4)	2.3 (2.2, 2.4)	0.004	0.28
In-hospital hypoglycemia occurrence (*n*, %)	Yes	291 (44.8%)	56 (54.4%)	0.088	0.19
	No	358 (55.2%)	47 (45.6%)		
Length of stay [M (P_25_, P_75_), day]		9.0 (7.0, 12.0)	9.0 (6.0, 11.0)	0.144	0.08

UEBMI, Urban Employee Basic Medical Insurance; URRBMI, Urban, Rural Resident Basic Medical Insurance; CSII, Continuous Subcutaneous Insulin Infusion; MDI, Multiple Daily Injections; LFIR, Low Frequency Insulin Regimen; PPGE, Postprandial Plasma Glucose Excursion; LAGE, Largest Amplitude of Glycemic Excursion; BMI, Body Mass Index; HbA1c, Glycated Hemoglobin A1c; C-peptide, Connecting peptide; eGFR, estimated Glomerular Filtration Rate; TC, Total Cholesterol; TG, Triglycerides; LDL-C, Low-Density Lipoprotein Cholesterol; HDL-C, High-Density Lipoprotein Cholesterol; RBC, Red Blood Cell; WBC, White Blood Cell; CRP, C-Reactive Protein; 25(OH)D, 25-hydroxyvitamin D; GAD, Glutamic Acid Decarboxylase antibody; ZnT8, Zinc Transporter 8 antibody; IAA, Insulin Autoantibody; IA-2, Insulinoma-Associated Antigen 2 antibody; ICA, Islet Cell Antibody; SMD, Standardized Mean Difference; Cr, Creatinine; U, Insulin unit; NA, Not Applicable.

Compared with patients without hypoglycemia in the derivation cohort, those with hypoglycemia had a lower male proportion, a lower BMI, longer diabetes duration, higher rates of previous hypoglycemia and insulin pump use, higher LAGE, and decreased fasting and postprandial C-peptide levels. Stratified analyses based on hypoglycemia status in the derivation and external validation cohorts are presented in **Supplementary Tables 5**, **6**.

**Figure 3:**
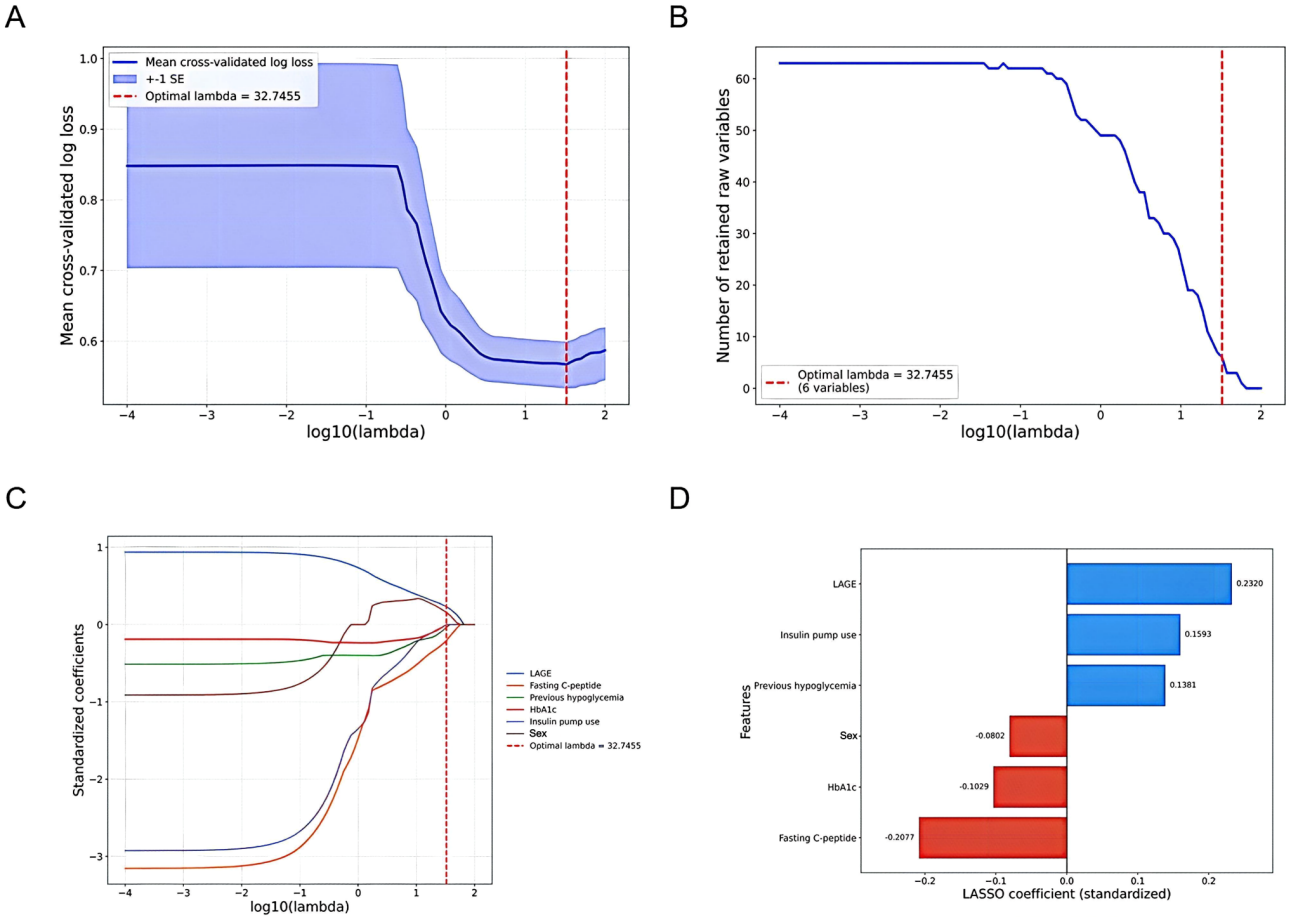
LASSO-based predictor selection in the training set. **(A)** Cross-validated mean log loss across the LASSO penalty path; the shaded area indicates ±1 SE. **(B)** Number of retained raw variables across the penalty path. **(C)** Coefficient trajectories of the retained predictors across log10(lambda). **(D)** Standardized non-zero coefficients at the selected lambda. The dashed vertical line indicates the selected penalty parameter (λ = 32.7455; log10 λ = 1.5152), chosen according to the prespecified rule of the best cross-validated model with no more than 6 raw variables. At this λ, 6 variables were retained: LAGE, fasting C-peptide, HbA1c, Sex, insulin pump use, and previous hypoglycemia.

Based on LASSO regression, six predictors were retained for the final predictive model: LAGE, fasting C-peptide, HbA1c, sex, insulin pump use, and previous hypoglycemia. VIF analysis confirmed no substantial multicollinearity, as the VIF value of each retained variable was below 5. The complete LASSO variable selection process and VIF results are shown in **Figure 3** and **Supplementary Figure 1**, respectively.

### 3.2 Model performance

Hyperparameter tuning was performed for logistic regression, random forest and XGBoost. The optimized hyperparameters for all three models are listed in **Supplementary Table 7**.

**Figure 4:**
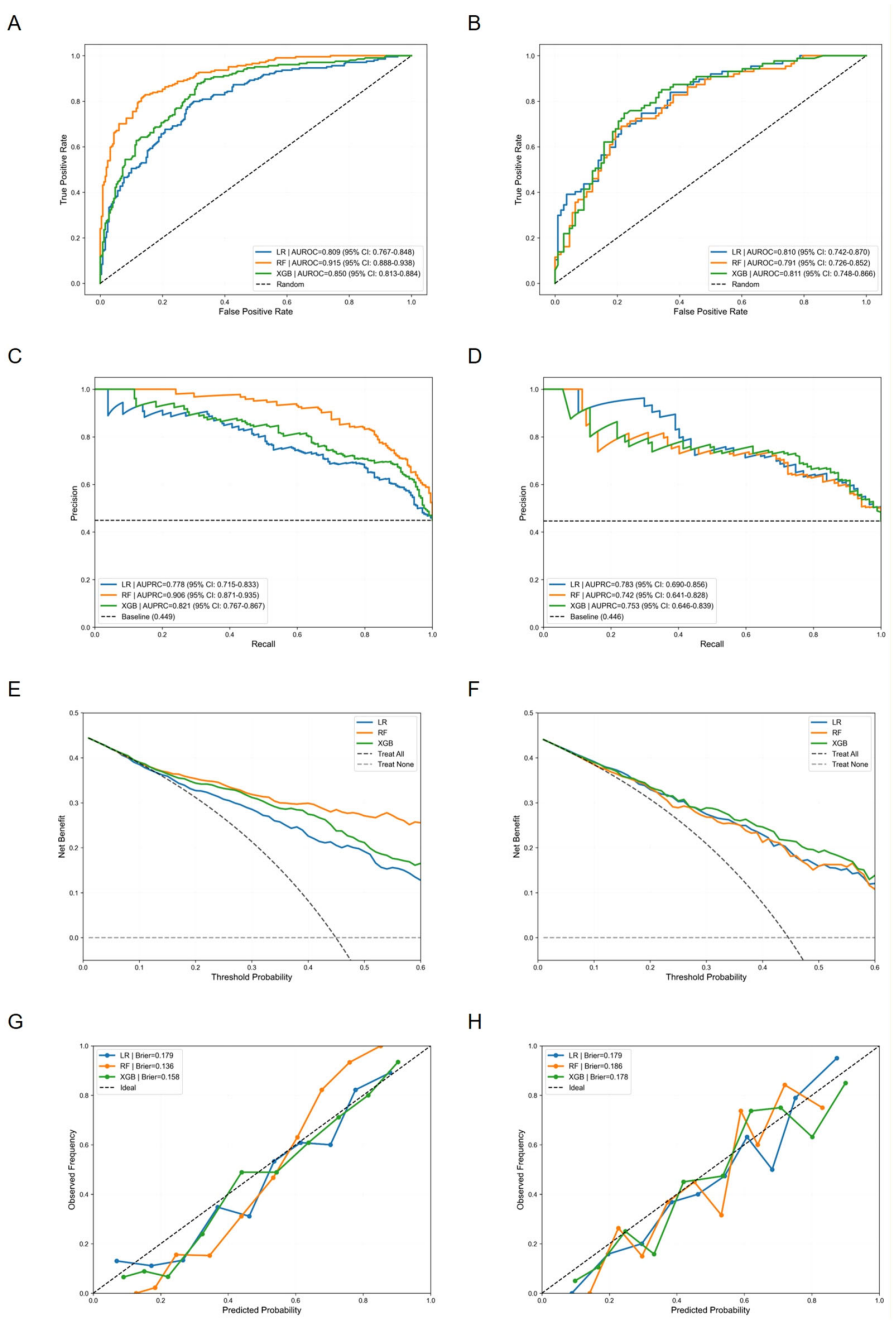
Model performance in the training and internal validation sets. **(A)** Receiver operating characteristic curve for the training set. **(B)** Receiver operating characteristic curve for the internal validation set. **(C)** Precision–recall curve for the training set. **(D)** Precision–recall curve for the internal validation set. **(E)** Decision curve analysis for the training set. **(F)** Decision curve analysis for the internal validation set. **(G)** Calibration curve for the training set. **(H)** Calibration curve for the internal validation set. All panels present results from logistic regression, random forest, and XGBoost models.

Model discrimination varied across datasets. In the training set, random forest yielded the highest AUROC (0.915, 95% *CI*: 0.888–0.938), followed by XGBoost (0.850, 95% *CI*: 0.813–0.884) and logistic regression (0.809, 95% *CI*: 0.767–0.848) (**Figure 4A**). In the internal validation set, XGBoost (0.811, 95% *CI*: 0.748–0.866) and logistic regression (0.810, 95% *CI*: 0.742–0.870) showed comparable discriminative performance, whereas random forest had a slightly lower AUROC of 0.791 (95% *CI*: 0.726–0.852) (**Figure 4B**). The AUROC differences among models in the internal validation set were small, ranging from 0.001 to 0.020. Although the 95% *CI*s of AUROC overlapped across the three models, pairwise DeLong tests showed statistically significant AUROC differences between logistic regression and random forest and between random forest and XGBoost, whereas no significant difference was observed between logistic regression and XGBoost (**Supplementary Table 8**).

For precision-recall performance, random forest obtained the highest AUPRC in the training set (**Figure 4C**), whereas all three models showed similar AUPRC values in the internal validation set (**Figure 4D**). Model calibration was evaluated via calibration curves and Brier scores. Random forest showed better calibration in the training set (Brier score = 0.136), while XGBoost had a relatively higher Brier score of 0.158. In the internal validation set, XGBoost presented the lowest Brier score and the closest alignment with the ideal calibration line. Decision curve analysis (DCA) demonstrated that all models provided greater net clinical benefit than the treat-all and treat-none strategies across a wide range of threshold probabilities (**Figures 4E, F**), and XGBoost achieved the highest net benefit in the internal validation cohort. Detailed performance metrics including AUROC, AUPRC and Brier scores are summarized in **Table 3**, and corresponding calibration curves are displayed in **Figures 4G, H**.

**Table 3:** Overall performance metrics of three prediction models across different datasets.

Dataset	Model	AUROC (95% *CI*)	AUPRC (95% *CI*)	Brier score
Training set	LR	0.809 (0.767–0.848)	0.778 (0.715–0.833)	0.179
Training set	RF	0.915 (0.888–0.938)	0.906 (0.871–0.935)	0.136
Training set	XGBoost	0.850 (0.813–0.884)	0.821 (0.767–0.867)	0.158
Internal validation	LR	0.810 (0.742–0.870)	0.783 (0.690–0.856)	0.179
Internal validation	RF	0.791 (0.726–0.852)	0.742 (0.641–0.828)	0.186
Internal validation	XGBoost	0.811 (0.748–0.866)	0.753 (0.646–0.839)	0.178
External validation	LR	0.753 (0.649–0.841)	0.758 (0.634–0.870)	0.206
External validation	RF	0.735 (0.631–0.826)	0.737 (0.608–0.844)	0.207
External validation	XGBoost	0.770 (0.668–0.855)	0.773 (0.654–0.874)	0.202

LR, logistic regression; RF, random forest; AUROC, area under the receiver operating characteristic curve; AUPRC, area under the precision-recall curve; lower Brier scores indicate better model calibration.

**Figure 5:**
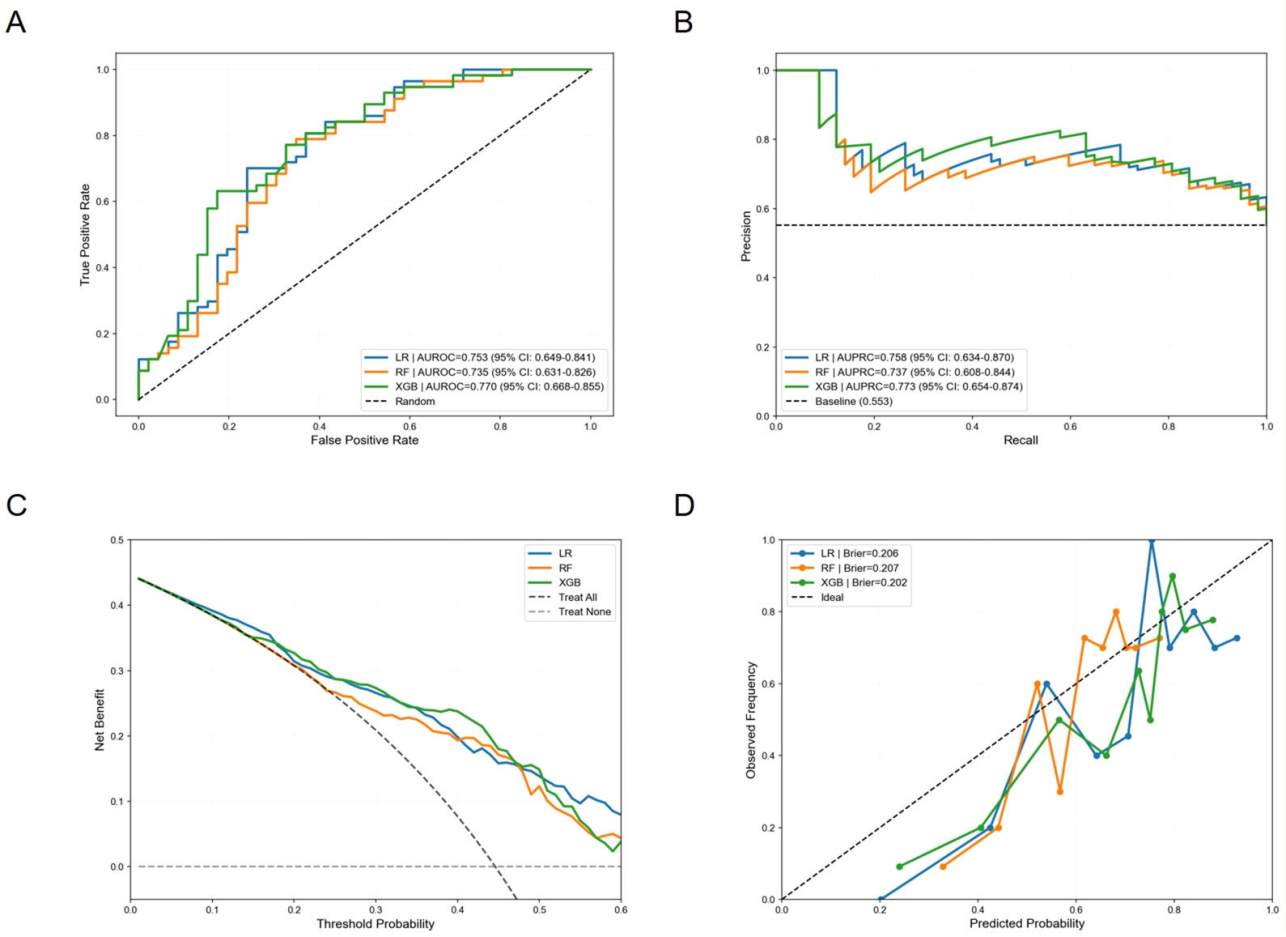
External validation of model performance. **(A)** Receiver operating characteristic curve. **(B)** Precision–recall curve. **(C)** Decision curve analysis. **(D)** Calibration curve. All panels show the performance of logistic regression, random forest, and XGBoost models in the external validation set.

In the external validation set, the three models had comparable discriminative performance, and pairwise DeLong tests confirmed no significant differences in AUROC among them (**Supplementary Table 8**). AUPRC and Brier scores were also similar across models in this cohort. All external validation results are visualized in **Figure 5**.

Considering comprehensive performance including discriminative ability, calibration, clinical net benefit and sensitivity at the predefined threshold, XGBoost was selected as the final model rather than choosing the model with the highest AUROC alone.

### 3.3 Threshold sensitivity, explainability, and subgroup performance

The optimal classification thresholds for each model were determined by maximizing the Youden index in the training set, and these cutoffs were applied uniformly to all validation cohorts. The optimal thresholds were 0.514 for logistic regression, 0.529 for random forest, and 0.495 for XGBoost. Threshold-dependent classification indicators are summarized in **Table 4**.

**Table 4:** Threshold-dependent classification performance and Brier scores of the prediction models.

Cohort	Model	Threshold	Accuracy	Sensitivity	Specificity	Precision	NPV	F1	MCC	Brier score
Training	LR	0.514	0.738	0.716	0.712	0.705	0.765	0.710	0.471	0.179
Training	RF	0.529	0.789	0.828	0.812	0.735	0.844	0.779	0.581	0.136
Training	XGBoost	0.495	0.786	0.789	0.768	0.749	0.820	0.768	0.571	0.158
Internal validation	LR	0.514	0.713	0.701	0.657	0.670	0.750	0.685	0.422	0.179
Internal validation	RF	0.529	0.723	0.759	0.722	0.667	0.781	0.710	0.450	0.186
Internal validation	XGBoost	0.495	0.723	0.701	0.735	0.685	0.755	0.693	0.441	0.178
External validation	LR	0.514	0.777	0.857	0.681	0.762	0.800	0.807	0.550	0.206
External validation	RF	0.529	0.767	0.839	0.681	0.758	0.780	0.797	0.529	0.207
External validation	XGBoost	0.495	0.786	0.857	0.702	0.774	0.805	0.814	0.569	0.202

LR, logistic regression; MCC, Matthews correlation coefficient; NPV, negative predictive value; RF, random forest; XGBoost, extreme gradient boosting. Brier scores were calculated based on predicted probabilities; lower values represent better calibration.

In the external validation set, threshold-dependent classification performance was generally comparable across the three models. XGBoost had balanced operating characteristics, with marginally higher accuracy, specificity, negative predictive value, F1 score, and Matthews correlation coefficient than logistic regression and random forest. Logistic regression yielded similar sensitivity, while random forest recorded lower sensitivity. These results indicate that the threshold defined in the training set for XGBoost maintained stable generalizability in the external cohort. Threshold-dependent metrics should be interpreted together with threshold-independent metrics and calibration.

**Figure 6:**
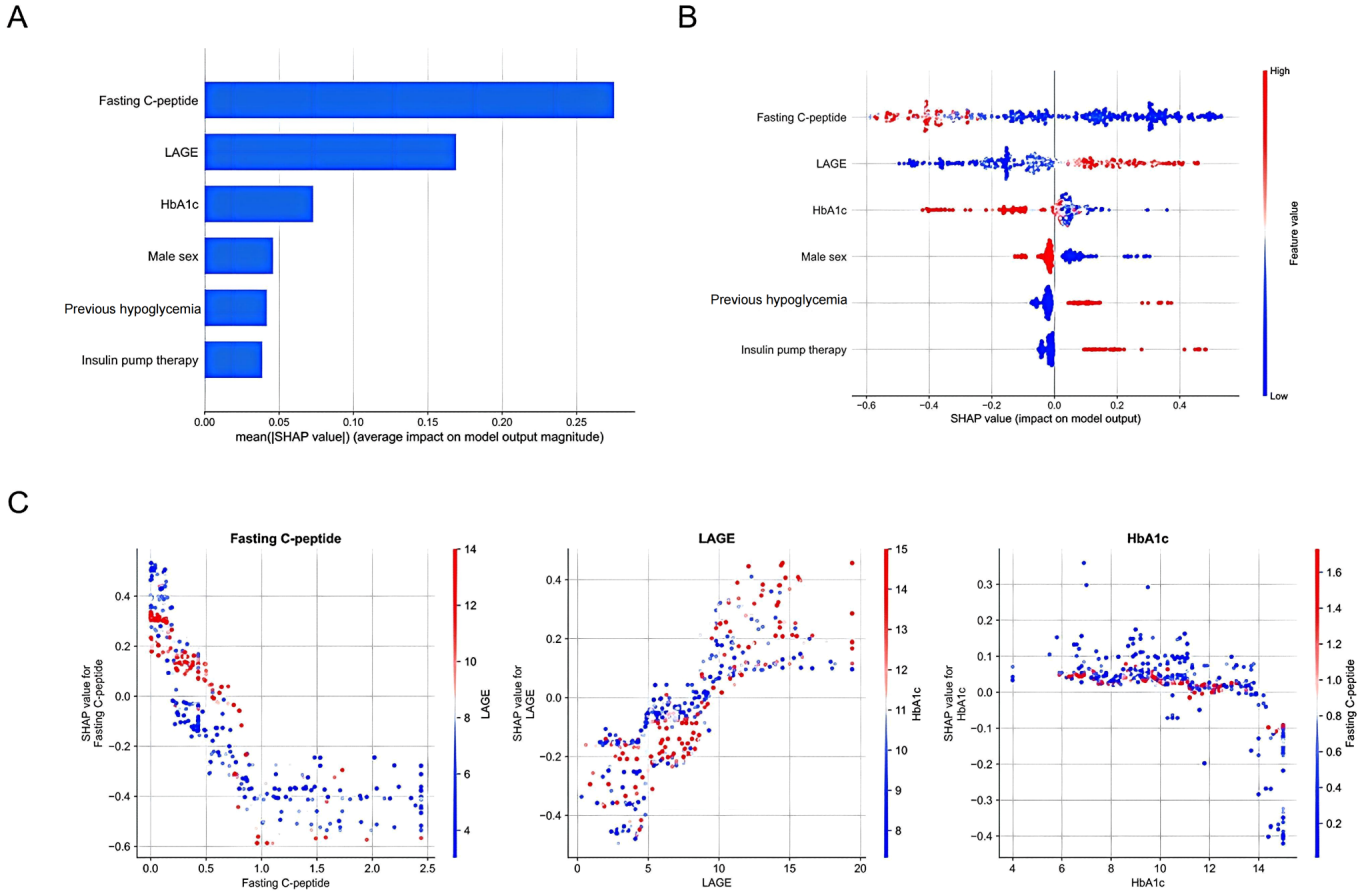
SHAP-based interpretation of the XGBoost model. **(A)** Mean absolute SHAP values showing global feature importance. **(B)** SHAP summary plot presenting the distribution and directional effect of all predictors. **(C)** SHAP dependence plots for fasting C-peptide, LAGE, and HbA1c. Higher fasting C-peptide was associated with lower predicted risk, whereas higher LAGE was associated with higher predicted risk. HbA1c showed a weaker and non-linear contribution to model predictions.

SHAP analysis was applied to quantify feature importance and enhance the interpretability of the final XGBoost model. Mean absolute SHAP values identified fasting C-peptide as the most influential predictor, followed by LAGE and HbA1c. Higher fasting C-peptide was associated with lower hypoglycemia risk, while elevated LAGE corresponded to increased risk. HbA1c exerted a relatively weak and non-linear effect on model predictions. Detailed SHAP analysis results are shown in **Figure 6**.

Multiple sensitivity analyses verified the robustness of the final XGBoost model. Threshold sensitivity analysis was conducted within a clinically reasonable range of 0.30 to 0.70, covering common cutoffs for clinical risk stratification. Model performance remained stable across this entire interval.

Leave-one-predictor analysis showed that removing LAGE only slightly reduced the external AUROC from 0.770 to 0.763, with negligible changes in Brier score and F1 score (**Supplementary Table 9**). By contrast, excluding fasting C-peptide led to a noticeable decline in discrimination, which highlighted its critical role in model prediction.

Further sensitivity analyses were performed using alternative missing-data handling approaches. Variable distributions remained stable before and after multiple imputation in both training and external validation datasets (**Supplementary Tables 3**, **4**). Analyses using median imputation, missing-indicator method, complete-case analysis and KNN imputation all yielded consistent results, with external AUROC ranging from 0.742 to 0.766 (**Supplementary Table 10**).

Additional analyses were performed among participants with complete islet autoantibody data to explore the incremental value of autoantibody markers. Adding GAD, IAA, ZnT8, IA-2 and ICA to the original six predictors did not improve model performance. The AUROC remained nearly unchanged in both the internal (0.781 to 0.770) and external (0.711 to 0.710) subsets. AUPRC and Brier scores were also comparable between the original and expanded models (**Supplementary Table 11**). In this limited antibody-complete subset, adding islet autoantibodies did not show measurable improvement in model performance. Given the small sample size and heterogeneous antibody availability, these findings should be interpreted as exploratory.

**Figure 7:**
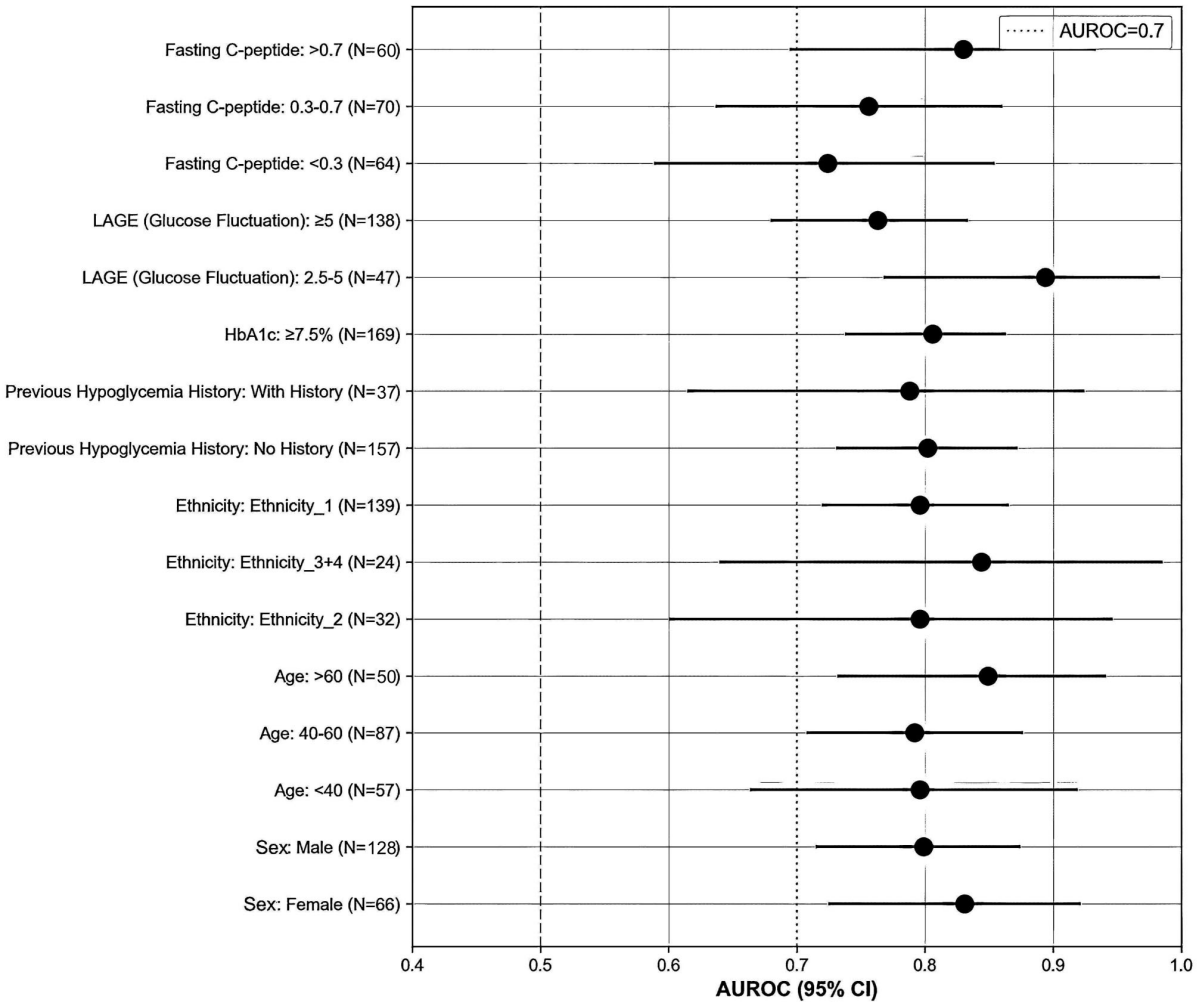
AUROC of the final model across predefined subgroups in the internal validation set. Points represent subgroup-specific AUROC estimates, and horizontal lines represent 95% CIs. Wide CIs in small subgroups indicated large estimation uncertainty. The dotted vertical line marks the reference AUROC of 0.7, and the dashed vertical line marks AUROC = 0.5.

Subgroup analyses were conducted in the internal validation set (*n* = 194) to assess model performance across clinically relevant strata. The model maintained acceptable discriminative ability in most subgroups. However, several subgroups defined by insulin pump use and fasting C-peptide levels had small sample sizes and limited event counts. Extreme values of specificity and positive predictive value observed in these small subgroups were considered artifacts caused by insufficient samples. Therefore, subgroup results should be interpreted with caution, and these analyses are regarded as exploratory. Detailed subgroup metrics are listed in **Supplementary Table 12**, and subgroup-level AUROC estimates are visualized in **Figure 7**.

## 4 Discussion

### 4.1 Principal findings

In this multicenter cohort of hospitalized patients with LADA, the overall incidence of in-hospital hypoglycemia was relatively high, which highlighted the necessity of individualized risk stratification for inpatients. Logistic regression, random forest and XGBoost exhibited comparable performance across validation datasets. XGBoost was selected as the final prediction model based on its acceptable discriminative ability, potential clinical utility in decision curve analysis, and relatively balanced threshold-dependent performance at the predefined threshold. All six predictors identified in this study were derived from routine laboratory tests and electronic medical records, requiring no invasive or expensive examinations. This characteristic enabled wide application in general inpatient settings.

Furthermore, SHAP analysis was adopted to provide a model interpretation framework consistent with pathophysiological mechanisms. It quantified the contribution of each variable to hypoglycemia risk, partially mitigated the black-box nature of machine learning models, and improved the clinical interpretability of this LADA-specific hypoglycemia prediction model.

### 4.2 Interpretation of predictive variables

To clarify the clinical mechanisms underlying predictors for in-hospital hypoglycemia among LADA patients, all variables were interpreted in combination with the core pathological features of LADA, including progressive pancreatic β-cell dysfunction and gradual decline of islet reserve (35).

Fasting C-peptide was identified as the strongest predictor of in-hospital hypoglycemia, and lower C-peptide levels corresponded to higher hypoglycemia risk. This finding was consistent with the natural progression of LADA. Persistent autoimmune-mediated injury may progressively impair pancreatic β-cells function, reduce endogenous insulin secretion, and weaken islet reserve (36). As a reliable biomarker reflecting basal islet secretory capacity, fasting C-peptide determined the ability to maintain glucose homeostasis and buffer glycemic fluctuations (37). Patients with impaired C-peptide secretion tended to have unstable glycemic control (38). Combined with inpatient stress, irregular food intake, and medication modification, these patients were more vulnerable to abrupt glucose decline and subsequent hypoglycemia (39). Clinically, fasting C-peptide can be used for routine risk screening to stratify hypoglycemia risk and guide targeted prevention among hospitalized LADA patients.

Islet autoantibodies were excluded from the primary model during LASSO variable selection. Sensitivity analyses restricted to participants with complete antibody data showed that adding GAD, IAA, ZnT8, IA-2 and ICA did not show measurable improvement in AUROC, AUPRC and Brier score. Although these immune markers did not show measurable incremental predictive value in the present cohort, they remained essential for confirming the autoimmune etiology of LADA (40). Considering their significance for disease classification, further prospective studies with standardized antibody detection are needed to explore whether antibody profiles can refine hypoglycemia risk prediction or identify immune-related high-risk subgroups.

LAGE, a key indicator of glycemic variability, was associated with increased risk of in-hospital hypoglycemia in the final model. SHAP dependence plots confirmed that elevated LAGE was associated with increased hypoglycemia risk. Higher LAGE reflected severe glucose excursions and impaired glucose regulation (7). Progressive insulin deficiency in LADA further undermined the body’s capacity to stabilize blood glucose, leading to more obvious glycemic lability compared with patients with T2DM (41). Multiple inpatient stressors, including physical exhaustion, irregular diet, and intensified antidiabetic therapy, further exacerbated glycemic variability. Excessive glucose fluctuation raised the risk of unexpected hypoglycemia, followed by neurological discomfort, falls and cardiovascular complications (42). Notably, LAGE can be calculated simply based on routine bedside glucose monitoring without extra tests. Incorporating this glycemic variability indicator may partially capture the metabolic instability of LADA, and improve the practical value of inpatient hypoglycemia risk stratification.

HbA1c reflects average blood glucose levels over the past two to three months and serves as a core indicator of long-term glycemic control (43). In LADA patients with low HbA1c, long-term intensive glycemic control was usually accompanied by progressive β-cell failure and insufficient endogenous insulin supply (44). The impaired glucose buffering capacity made these patients susceptible to hypoglycemia during hospitalization, especially when diet or medication was adjusted (45). In addition, long-term strict glycemic management may lead to unrecognized asymptomatic hypoglycemia, which was more likely to be detected during intensive inpatient monitoring and increased the recorded incidence of hypoglycemia (44). In contrast, patients with HbA1c above 9.0% generally had poor long-term glycemic control. Inpatient management for this group focused on gradual correction of hyperglycemia, resulting in a relatively low baseline risk of hypoglycemia (46). Even so, clinicians should adjust antidiabetic medication doses slowly and individually for these patients to avoid iatrogenic hypoglycemia caused by aggressive treatment.

Sex differences were closely linked to hypoglycemia susceptibility in hospitalized LADA patients. Distinct physiological characteristics, metabolic profiles and disease management patterns jointly led to varied hypoglycemia risks between sexes (47). In line with previous evidence, SHAP analysis indicated a higher hypoglycemia risk in female patients, and subgroup analyses further verified the stable predictive performance of the model in female populations (48). Fluctuations in estrogen and progesterone can disrupt glucose regulation (49). On the basis of inherent β-cell dysfunction in LADA, hormonal imbalance further aggravated glycemic lability (40). In addition, females usually had lower body weight and higher insulin sensitivity, which increased their vulnerability to hypoglycemia (50). Excessive dietary restriction and irregular self-adjustment of medication, driven by overconcerns about glycemic control, also contributed to the higher hypoglycemia incidence among female patients (51). Overall, female LADA patients with low body weight and reduced fasting C-peptide represent a key high-risk inpatient subgroup, who require more frequent blood glucose monitoring and individualized conservative anti-hypoglycemia regimens.

Insulin pump use was significantly associated with elevated in-hospital hypoglycemia risk in LADA patients, which may indicate more severe disease and poorer islet function among pump users (52). While insulin pumps enable flexible insulin delivery and mitigate chronic glycemic variability (53), delayed dose adjustment for changes in diet or physical activity, as well as equipment malfunction, can trigger acute hypoglycemia (54). Therefore, hospitalized LADA patients using insulin pumps require real-time glucose monitoring, regular equipment inspection and timely individualized dose adjustment to prevent hypoglycemia-related adverse events.

Previous hypoglycemia was another important predictor of in-hospital hypoglycemia in LADA patients, and SHAP analysis suggested its contribution to individualized risk assessment. A history of recurrent hypoglycemia may reflect impaired β-cell reserve, unstable glycemic regulation, and increased vulnerability to subsequent glucose decline. Repeated hypoglycemic episodes further impaired glucose adaptability and increased recurrence risk (55). Meanwhile, recurrent hypoglycemia may impair hypoglycemia awareness, reduce early symptom perception and raise the probability of severe hypoglycemia (56). Hypoglycemia-related anxiety may also lead to excessive dietary restriction and non-standard medication use, further destabilizing blood glucose during hospitalization (57). Accordingly, LADA patients with a history of hypoglycemia should be classified as a high-risk group, requiring targeted dynamic monitoring and personalized preventive interventions during hospitalization.

### 4.3 Clinical implications and translational value

Subgroup analyses showed that the model maintained stable performance across major clinical strata in the internal validation cohort. Nevertheless, these results should be interpreted with caution due to small sample sizes, limited outcome events, wide confidence intervals and unstable point estimates in several subgroups. Extreme values observed in individual small subgroups were regarded as statistical bias rather than superior model efficacy. Therefore, subgroup results were only used to assess the internal consistency of model discrimination, instead of identifying optimal subgroups or proving generalizability to external populations.

From the perspective of clinical translation, the potential net clinical benefit within the risk threshold of 0.2–0.4 supports the possible application of this model for stratifying moderate-to-high hypoglycemia risk among hospitalized LADA patients. Under limited medical resources, this prediction tool helps clinicians identify high-risk patients and implement targeted interventions, including intensified glucose testing, nocturnal monitoring and optimized insulin regimens (58).

Random forest showed relatively high sensitivity in some validation settings, whereas XGBoost demonstrated relatively balanced validation performance, potential clinical net benefit, acceptable sensitivity, and improved interpretability. Therefore, XGBoost was considered a reasonable pragmatic choice for further clinical evaluation rather than a model with proven superiority across all metrics (59). XGBoost was finally selected for its overall balanced performance, as no single algorithm demonstrated consistent statistical superiority across all evaluation metrics.

### 4.4 Study limitations and future perspectives

Several limitations of this study should be acknowledged. First, the retrospective multicenter design carries inherent selection bias. Heterogeneous glucose monitoring protocols and treatment strategies across participating centers may affect the detection of hypoglycemic episodes. Cases and controls had asymmetric overall follow-up periods, and individual counts of valid glucose measurements could not be retrieved from original electronic medical records. Despite standardized calculation rules, differences in glucose monitoring frequency across patients could not be fully excluded, and the shared glucose source for glycemic indicators and hypoglycemic events may still raise concerns regarding residual outcome leakage and circularity. Second, all predictors were selected via LASSO logistic regression. This linear approach cannot capture nonlinear relationships and interactions among variables, which may compromise the performance of tree-based models including random forest and XGBoost. Third, a 40% missing rate threshold was applied during the screening of candidate variables. Although this cutoff is supported by existing literature and widely adopted in clinical prediction research, it may still introduce methodological bias. Islet autoantibodies were excluded from the primary model during LASSO variable selection due to their poor predictive performance. Their limited routine availability across participating centers further justified this exclusion. Additional analyses among participants with complete antibody data did not show measurable improvement in overall model performance after adding these markers. Fourth, all subgroup analyses were performed solely on the internal validation cohort. Several subgroups had small sample sizes and limited outcome events, leading to wide confidence intervals and unstable results. These subgroup findings are exploratory in nature and cannot be generalized to external populations. Fifth, pairwise DeLong tests were only conducted for AUROC, while no paired tests were performed for AUPRC and Brier score. Classification thresholds determined from the training set were applied to all validation cohorts without adjustment. Differences in hypoglycemic event rates across datasets may have affected the transportability of fixed training-derived thresholds, and threshold-dependent classification metrics should therefore be interpreted cautiously.

Future research should conduct large-sample prospective multicenter studies to reduce retrospective bias and enhance model generalizability. Algorithm-adapted feature selection methods are recommended to maximize the strengths of tree-based models. In addition, comprehensive statistical tests for all predictive metrics and cohort-specific threshold recalibration will help improve real-world applicability. Standardized protocols for islet autoantibody testing should be implemented in subsequent prospective research, to clarify whether LADA immune markers can add incremental value to hypoglycemia risk prediction. Enlarging sample sizes of high-risk subgroups will also yield more robust analytical findings. Moreover, developing and prospectively validating dynamic early warning models based on fixed admission time windows can facilitate routine clinical use. Continued external validation and iterative optimization are still required to promote the clinical application of this prediction tool for hospitalized patients with LADA.

## 5 Conclusions

This study developed an interpretable XGBoost model to predict in-hospital hypoglycemia in patients with LADA. All predictors incorporated into the model were derived from routine laboratory tests and electronic medical records, ensuring wide applicability in clinical practice. The model showed acceptable discrimination and calibration, and decision curve analysis suggested potential net clinical benefit for risk stratification. Compared with logistic regression and random forest, XGBoost showed balanced overall performance and improved interpretability with SHAP analysis, which helped partially mitigate the black-box nature of machine learning models. Further prospective multicenter validation and external recalibration are required to improve its generalizability prior to widespread clinical application.

## Data Availability

Due to patient privacy protection and restrictions imposed by institutional ethics approval, the individual-level clinical data used in this study are not publicly available. De-identified data supporting the findings of this study may be made available from the corresponding author upon reasonable request, subject to institutional ethics approval and a data use agreement. The analysis code required to reproduce the main analyses may also be made available from the corresponding author upon reasonable request for academic and non-commercial purposes, subject to institutional approval. Requests to access the datasets should be directed to Jianjun Zou, zoujianjun100@126.com; Xiaoli Zhu, zhugu2012@163.com.
