# Supplementary material for: Explainable machine learning model for in-hospital hypoglycemia risk in patients with latent autoimmune diabetes in adults
Source: Front Immunol. 2026 Jul 27;17:1870438. doi: 10.3389/fimmu.2026.1870438 (PMC13454309; doi:10.3389/fimmu.2026.1870438)
Supplement: Supplementary file 1 [file Table1.docx]

Supplementary Figure S1 VIF for predictors retained in the final model

Variance inflation factors were calculated to assess multicollinearity among selected predictors. All values were below 5. Dashed vertical lines represent the reference thresholds of 5 and 10.

Supplementary Table S1 Full inventory of study variables, operational definitions, measurement timing, and pre-imputation missingness in the derivation and external validation cohorts

| **Variable** | **Domain** | **Definition** | **Measurement timing** | **Missing rate (D), %** | **Missing rate (E), %** |
| --- | --- | --- | --- | --- | --- |
| In-hospital hypoglycemia occurrence | Primary outcome | In-hospital hypoglycemia during index hospitalization | Outcome during index hospitalization | 0.00 | 0.00 |
| First hypoglycemia glucose value | Event descriptor / outcome-adjacent variable | Glucose value at the first hypoglycemic event during index hospitalization | At the first in-hospital hypoglycemic event; not eligible for predictor derivation | 55.32 | 56.31 |
| Sex | Demographic | Biological sex recorded in the electronic health record | At admission | 0.00 | 0.00 |
| Age | Demographic | Age at index hospitalization, years | At admission | 0.00 | 0.00 |
| Marital status | Demographic / socioeconomic | Marital status | At admission | 0.00 | 0.00 |
| Ethnicity | Demographic | Self-reported ethnicity | Historical information recorded at admission | 0.00 | 0.00 |
| Education level | Socioeconomic | Education level category | Historical information recorded at admission | 0.00 | 0.00 |
| Insurance status | Socioeconomic / administrative | Insurance type or coverage status | At admission | 0.00 | 0.00 |
| BMI | Anthropometric | Body mass index, kg/m² | First available measurement during index hospitalization, typically at admission | 4.16 | 0.97 |
| Length of stay | Secondary outcome | Length of stay during index hospitalization | From admission to discharge | 0.00 | 0.00 |
| Smoking status | Lifestyle | Current or past smoking status | Historical information recorded at admission | 0.00 | 0.00 |
| Duration of smoking | Lifestyle | Duration of smoking exposure, years | Historical information recorded at admission | 53.31 | 33.98 |
| Alcohol use | Lifestyle | Current or past alcohol use | Historical information recorded at admission | 0.00 | 0.00 |
| Duration of alcohol use | Lifestyle | Duration of alcohol exposure, years | Historical information recorded at admission | 74.11 | 52.43 |
| Age at diabetes onset | Disease history | Age at diabetes / LADA onset | Historical information recorded at admission | 0.00 | 58.25 |
| Diabetes duration | Disease history | Duration of diabetes / LADA, years | Historical information recorded at admission | 0.00 | 0.00 |
| Previous hypoglycemia | Disease history | Prior history of hypoglycemia before index hospitalization | Historical information recorded at admission | 4.16 | 0.00 |
| Family history of diabetes | Family history | Family history of diabetes mellitus | Historical information recorded at admission | 0.00 | 0.00 |
| History of hypertension | Medical history | History of hypertension | Historical information recorded at admission | 0.00 | 0.00 |
| Ketosis | Acute metabolic state | Ketosis present at admission or documented before reference time | At admission or before reference time | 0.00 | 0.00 |
| Diabetic ketoacidosis | Acute metabolic state | Diabetic ketoacidosis present at admission or documented before reference time | At admission or before reference time | 30.05 | 0.00 |
| Diabetic retinopathy | Diabetes complication | History of diabetic retinopathy | Historical diagnosis present at or before admission | 0.00 | 0.00 |
| Diabetic kidney disease | Diabetes complication | History of diabetic kidney disease | Historical diagnosis present at or before admission | 0.00 | 0.00 |
| Diabetic neuropathy | Diabetes complication | History of diabetic neuropathy | Historical diagnosis present at or before admission | 0.00 | 0.00 |
| Diabetic vascular disease | Diabetes complication | History of diabetic vascular disease | Historical diagnosis present at or before admission | 0.00 | 0.97 |
| Diabetic foot | Diabetes complication | History of diabetic foot | Historical diagnosis present at or before admission | 0.00 | 0.00 |
| Number of diabetic complications | Comorbidity burden | Count of diabetes-related complications | Historical information available at admission | 0.15 | 0.00 |
| Sleep disorder | Medical history | History of sleep disorder | Historical information recorded at admission | 0.00 | 0.00 |
| Polypharmacy | Treatment / medication burden | Use of multiple medications according to the prespecified study definition | Medication exposure before reference time | 0.00 | 0.00 |
| Oral hypoglycemic agent use | Treatment exposure | Use of oral hypoglycemic agents before reference time | Medication exposure before reference time | 0.00 | 0.00 |
| Insulin regimen | Treatment exposure | Inpatient insulin regimen category | Recorded before reference time | 4.93 | 0.00 |
| Insulin pump use | Treatment exposure | Insulin pump use during index hospitalization before reference time | Recorded before reference time | 0.15 | 0.00 |
| Total daily insulin dose | Treatment exposure | Total daily insulin dose during index hospitalization before reference time | Recorded before reference time | 4.47 | 1.94 |
| Premeal insulin use | Treatment exposure | Premeal or bolus insulin use | Recorded before reference time | 4.47 | 1.94 |
| Basal insulin use | Treatment exposure | Basal insulin use or basal insulin dose | Recorded before reference time | 4.31 | 1.94 |
| Insulin type | Treatment exposure | Type of insulin used | Recorded before reference time | 4.31 | 1.94 |
| Postprandial glucose excursion | Dynamic glycemic metric | Postprandial glucose excursion derived from eligible pre-reference glucose records | Derived from glucose records before reference time | 26.96 | 27.18 |
| Largest amplitude of glycemic excursion | Dynamic glycemic metric | Largest amplitude of glycemic excursion derived from eligible pre-reference glucose records | Derived from glucose records before reference time only | 0.00 | 7.77 |
| Mean blood glucose | Dynamic glycemic metric | Mean blood glucose derived from eligible pre-reference glucose records | Derived from glucose records before reference time only | 0.00 | 0.00 |
| HbA1c | Laboratory / glycemic control | Glycated hemoglobin | First available laboratory test before reference time | 2.31 | 1.94 |
| Fasting C-peptide | Laboratory / β-cell function | Fasting C-peptide concentration | First available fasting test before reference time | 11.09 | 4.85 |
| 0.5-h C-peptide | Laboratory / β-cell function | 0.5-hour C-peptide after stimulation or meal | First available test before reference time | 44.07 | 4.85 |
| 1-h C-peptide | Laboratory / β-cell function | 1-hour C-peptide after stimulation or meal | First available test before reference time | 39.91 | 5.83 |
| 2-h C-peptide | Laboratory / β-cell function | 2-hour C-peptide after stimulation or meal | First available test before reference time | 29.58 | 4.85 |
| Serum creatinine | Laboratory / renal function | Serum creatinine | First available laboratory test before reference time | 1.08 | 0.00 |
| eGFR | Laboratory / renal function | Estimated glomerular filtration rate derived from serum creatinine | Derived from first available creatinine before reference time | 1.39 | 0.00 |
| Total protein | Laboratory / biochemistry | Total protein | First available laboratory test before reference time | 4.78 | 0.00 |
| Total cholesterol | Laboratory / lipid profile | Total cholesterol | First available laboratory test before reference time | 2.77 | 2.91 |
| Triglycerides | Laboratory / lipid profile | Triglycerides | First available laboratory test before reference time | 2.77 | 2.91 |
| LDL-C | Laboratory / lipid profile | Low-density lipoprotein cholesterol | First available laboratory test before reference time | 2.93 | 2.91 |
| HDL-C | Laboratory / lipid profile | High-density lipoprotein cholesterol | First available laboratory test before reference time | 2.93 | 2.91 |
| Hemoglobin | Laboratory / hematology | Hemoglobin | First available laboratory test before reference time | 0.92 | 0.00 |
| RBC count | Laboratory / hematology | Red blood cell count | First available laboratory test before reference time | 1.08 | 0.00 |
| WBC count | Laboratory / hematology / inflammation | White blood cell count | First available laboratory test before reference time | 1.23 | 0.00 |
| CRP | Laboratory / inflammation | C-reactive protein | First available laboratory test before reference time | 35.44 | 42.72 |
| 25-hydroxyvitamin D | Laboratory / micronutrient | Serum 25-hydroxyvitamin D | First available laboratory test before reference time | 64.1 | 7.77 |
| Urinary microalbumin | Laboratory / renal marker | Urinary microalbumin | First available laboratory test before reference time | 43.91 | 18.45 |
| Urinary protein | Laboratory / renal marker | Urinary protein | First available laboratory test before reference time | 24.96 | 0.00 |
| GAD antibody | Immunologic marker | Glutamic acid decarboxylase autoantibody | Baseline immunologic test available before reference time | 36.98 | 33.01 |
| ZnT8 antibody | Immunologic marker | Zinc transporter 8 autoantibody | Baseline immunologic test available before reference time | 72.11 | 33.01 |
| IAA | Immunologic marker | Insulin autoantibody | Baseline immunologic test available before reference time | 36.67 | 33.01 |
| IA-2 antibody | Immunologic marker | Islet antigen-2 autoantibody | Baseline immunologic test available before reference time | 72.42 | 33.01 |
| ICA | Immunologic marker | Islet cell autoantibody | Baseline immunologic test available before reference time | 71.96 | 45.63 |
| Serum potassium | Laboratory / electrolyte | Serum potassium | First available laboratory test before reference time | 10.48 | 0.00 |
| Serum sodium | Laboratory / electrolyte | Serum sodium | First available laboratory test before reference time | 10.48 | 0.00 |
| Serum calcium | Laboratory / biochemistry | Serum calcium | First available laboratory test before reference time | 11.25 | 0.00 |

Table note: The raw study variable inventory comprised 66 variables. In-hospital hypoglycemia occurrence, length of stay, and first hypoglycemia glucose value were not eligible for candidate predictor screening because they represented the primary outcome, secondary outcome, and event descriptor, respectively. The remaining 63 variables formed the initial candidate predictor pool before missingness screening. Variables with > 40% missingness in the derivation cohort were excluded before imputation and LASSO-based feature selection, but were retained in this table to provide a complete overview of the raw data structure. Missing rates are reported before imputation. For inpatient time-varying variables, only data available before the corresponding reference time point were used for predictor derivation. Abbreviations: D, derivation cohort; E, external validation cohort; BMI, body mass index; CRP, C-reactive protein; HbA1c, glycated hemoglobin; HDL-C, high-density lipoprotein cholesterol; LDL-C, low-density lipoprotein cholesterol; LAGE, largest amplitude of glycemic excursion; PPGE, postprandial glucose excursion; IAA, insulin autoantibody; ICA, islet cell autoantibody; eGFR, estimated glomerular filtration rate; RBC, red blood cell; WBC, white blood cell.

Supplementary Table S2 Main software and package versions used for model development and interpretation

| **Software/package** | **Version** |
| --- | --- |
| SPSS | 25.0 |
| Python | 3.10.4 |
| scikit-learn | 1.4.0 |
| XGBoost | 1.5.1 |
| SHAP | 0.42.0 |
| Random seed | 42 |
| Optuna | 4.5.0 |

Table note: Only the main software and model-related packages used for statistical analysis, model development, and model interpretation are shown.

Supplementary Table S3 Comparison of variable distributions before and after imputation in the derivation cohort

| **Variable** | **SMD** | ***P* value** |
| --- | --- | --- |
| BMI | 0.0180 | 1.000 |
| Previous hypoglycemia | 0.0016 | 1.000 |
| Diabetic ketoacidosis | 0.1107 | 0.073 |
| Number of diabetic complications | 0.0009 | 0.990 |
| Insulin regimen | 0.0403 | 0.755 |
| Insulin pump use | 0.0005 | 1.000 |
| Total daily insulin dose | 0.0057 | 1.000 |
| Premeal insulin use | 0.0006 | 1.000 |
| Basal insulin use | 0.0092 | 1.000 |
| Insulin type | 0.0568 | 0.937 |
| PPGE | 0.0079 | 0.869 |
| HbA1c | 0.0010 | 1.000 |
| Fasting C-peptide | 0.0040 | 0.977 |
| 1-h C-peptide | 0.0223 | 0.605 |
| 2-h C-peptide | 0.0191 | 0.485 |
| Serum creatinine | 0.0024 | 1.000 |
| eGFR | 0.0084 | 1.000 |
| Total protein | 0.0173 | 1.000 |
| Total cholesterol | 0.0031 | 1.000 |
| Triglycerides | 0.0015 | 1.000 |
| LDL-C | 0.0007 | 1.000 |
| HDL-C | 0.0026 | 1.000 |
| Hemoglobin | 0.0006 | 1.000 |
| RBC count | 0.0007 | 1.000 |
| WBC count | 0.0004 | 1.000 |
| CRP | 0.0053 | 0.200 |
| Urinary protein | 0.0018 | 0.300 |
| GAD antibody | 0.0014 | 1.000 |
| IAA | 0.0673 | 0.316 |
| Serum potassium | 0.0100 | 1.000 |
| Serum sodium | 0.0061 | 0.991 |
| Serum calcium | 0.0201 | 0.999 |

Table note: This table compares variable distributions before and after imputation in the derivation cohort. For each variable, the pre-imputation distribution among observed values was compared with the post-imputation distribution in the completed dataset. Standardized mean differences (SMDs) were used as the primary measure of distributional comparability, and P values are provided as supplementary information. Smaller SMDs indicate less distributional shift after imputation; values < 0.1 were considered to indicate negligible imbalance. Abbreviations: SMD, standardized mean difference; PPGE, postprandial glucose excursion; HbA1c, glycated hemoglobin; eGFR, estimated glomerular filtration rate; LDL-C, low-density lipoprotein cholesterol; HDL-C, high-density lipoprotein cholesterol; RBC, red blood cell; WBC, white blood cell; CRP, C-reactive protein; GAD, glutamic acid decarboxylase; IAA, insulin autoantibody.

Supplementary Table S4 Comparison of variable distributions before and after imputation in the external validation cohort

| **Variable** | **SMD** | ***P* value** |
| --- | --- | --- |
| BMI | 0.0084 | 1.000 |
| Duration of smoking | 0.0488 | 0.619 |
| Diabetic vascular disease | 0.0030 | 1.000 |
| Total daily insulin dose | 0.0038 | 1.000 |
| Premeal insulin use | 0.0129 | 1.000 |
| Basal insulin use | 0.0034 | 1.000 |
| Insulin type | 0.0148 | 1.000 |
| PPGE | 0.0877 | 0.978 |
| LAGE | 0.0088 | 1.000 |
| HbA1c | 0.0081 | 1.000 |
| Fasting C-peptide | 0.0133 | 1.000 |
| 0.5-h C-peptide | 0.0216 | 1.000 |
| 1-h C-peptide | 0.0269 | 1.000 |
| 2-h C-peptide | 0.0168 | 1.000 |
| Total cholesterol | 0.0083 | 1.000 |
| Triglycerides | 0.0022 | 1.000 |
| LDL-C | 0.0121 | 1.000 |
| HDL-C | 0.0150 | 1.000 |
| 25-hydroxyvitamin D | 0.0112 | 1.000 |
| Urinary microalbumin | 0.1513 | 0.589 |
| GAD antibody | 0.0137 | 1.000 |
| ZnT8 antibody | 0.0347 | 0.160 |
| IAA | 0.0480 | 0.857 |
| IA-2 antibody | 0.2077 | 0.224 |

Table note: This table compares variable distributions before and after imputation in the external validation cohort. For each variable, the pre-imputation distribution among observed values was compared with the post-imputation distribution in the completed dataset. Standardized mean differences (SMDs) were used as the primary measure of distributional comparability, and P values are provided as supplementary information. Smaller SMDs indicate less distributional shift after imputation; values < 0.1 were considered to indicate negligible imbalance. Abbreviations. SMD, standardized mean difference; PPGE, postprandial glucose excursion; HbA1c, glycated hemoglobin; LDL-C, low-density lipoprotein cholesterol; HDL-C, high-density lipoprotein cholesterol; GAD, glutamic acid decarboxylase; ZnT8, zinc transporter 8; IAA, insulin autoantibody.

Supplementary Table S5 Baseline characteristics stratified by hypoglycemia status in the development cohort

| **Variable** | | **No hypoglycemia**  **(*n* = 358)** | **Hypoglycemia**  **(*n* = 291)** | ***P* value** | **SMD** |
| --- | --- | --- | --- | --- | --- |
| Sex (*n*, %) | Male | 237 (66.2%) | 163 (56.0%) | 0.009 | 0.21 |
|  | Female | 121 (33.8%) | 128 (44.0%) |  |  |
| Age [M (P_25_, P_75_), year] | | 50.0 (37.0, 60.0) | 51.0 (43.0, 60.0) | 0.116 | 0.14 |
| Marital status (*n*, %) | Married | 303 (84.6%) | 247 (84.9%) | 0.615 | 0.08 |
|  | Unmarried | 36 (10.1%) | 23 (7.9%) |  |  |
|  | Divorced | 13 (3.6%) | 14 (4.8%) |  |  |
|  | Widowed | 6 (1.7%) | 7 (2.4%) |  |  |
| Ethnicity (*n*, %) | Han | 273 (76.3%) | 179 (61.5%) | < 0.001 | 0.32 |
|  | Bai | 48 (13.4%) | 68 (23.4%) |  |  |
|  | Yi | 18 (5.0%) | 25 (8.6%) |  |  |
|  | Other minorities | 19 (5.3%) | 19 (6.5%) |  |  |
| Education level (*n*, %) | Illiterate/Primary school education | 82 (22.9%) | 93 (32.0%) | 0.032 | 0.20 |
|  | Junior / Senior high school | 185 (51.7%) | 135 (46.4%) |  |  |
|  | University and above | 91 (25.4%) | 63 (21.6%) |  |  |
| Insurance status (*n*, %) | UEBMI | 158 (44.1%) | 115 (39.5%) | 0.022 | 0.18 |
|  | URRBMI | 184 (51.4%) | 172 (59.1%) |  |  |
|  | Self-payment | 16 (4.5%) | 4 (1.4%) |  |  |
| BMI [M (P_25_, P_75_), kg/m^2^] | | 21.7 (20.0, 23.8) | 20.6 (18.9, 22.9) | < 0.001 | 0.29 |
| Smoking status (*n*, %) | Yes | 179 (50.0%) | 133 (45.7%) | 0.305 | 0.09 |
|  | No | 179 (50.0%) | 214 (54.3%) |  |  |
| Duration of smoking [M (P_25_, P_75_), year] | | 26.0 (16.0, 34.3) | 26.0 (20.0, 32.0) | 0.882 | 0.00 |
| Alcohol use (*n*, %) | Yes | 99 (27.7%) | 77 (26.5%) | 0.790 | 0.03 |
|  | No | 259 (72.3%) | 214 (73.5%) |  |  |
| Duration of alcohol use [M (P25, P75), year] | | 20.6 (14.1, 30.0) | 22.0 (16.8, 31.1) | 0.049 | 0.15 |
| Age at diabetes onset [M (P25, P75), year] | | 45.0 (34.0, 54.0) | 45.0 (35.0, 53.5) | 0.677 | 0.02 |
| Diabetes duration [M (P25, P75), year] | | 2.0 (0.0, 7.0) | 5.0 (1.0, 10.0) | < 0.001 | 0.30 |
| Previous hypoglycemia (*n*, %) | Yes | 36 (10.1%) | 75 (25.8%) | < 0.001 | 0.41 |
|  | No | 322 (89.9%) | 216 (74.2%) |  |  |
| Family history of diabetes (*n*, %) | Yes | 67 (18.7%) | 46 (15.8%) | 0.350 | 0.08 |
|  | No | 291 (81.3%) | 245 (84.2%) |  |  |
| History of hypertension (*n*, %) | Yes | 73 (20.4%) | 58 (19.9%) | 0.922 | 0.01 |
|  | No | 285 (79.6%) | 233 (80.1%) |  |  |
| Ketosis (*n*, %) | Yes | 132 (36.9%) | 115 (39.5%) | 0.516 | 0.05 |
|  | No | 226 (63.1%) | 176 (60.5%) |  |  |
| Diabetic ketoacidosis (*n*, %) | Yes | 50 (14.0%) | 37 (12.7%) | 0.728 | 0.04 |
|  | No | 308 (86.0%) | 254 (87.3%) |  |  |
| Diabetic retinopathy (*n*, %) | Yes | 60 (16.8%) | 56 (19.2%) | 0.412 | 0.06 |
|  | No | 298 (83.2%) | 235 (80.8%) |  |  |
| Diabetic kidney disease (*n*, %) | Yes | 31 (8.7%) | 36 (12.4%) | 0.153 | 0.12 |
|  | No | 327 (91.3%) | 255 (87.6%) |  |  |
| Diabetic neuropathy (*n*, %) | Yes | 240 (67.0%) | 222 (76.3%) | 0.011 | 0.21 |
|  | No | 118 (33.0%) | 69 (23.7%) |  |  |
| Diabetic vascular disease (*n*, %) | Yes | 89 (24.9%) | 92 (31.6%) | 0.065 | 0.15 |
|  | No | 269 (75.1%) | 199 (68.4%) |  |  |
| Diabetic foot (*n*, %) | Yes | 4 (1.1%) | 2 (0.7%) | 0.696 | 0.05 |
|  | No | 354 (98.9%) | 289 (99.3%) |  |  |
| Number of diabetic complications [M (P_25_, P_75_)] | | 2.0 (1.0, 2.0) | 2.0 (1.0, 3.0) | 0.002 | 0.23 |
| Sleep disorder (*n*, %) | Yes | 10 (2.8%) | 9 (3.1%) | 0.820 | 0.02 |
|  | No | 348 (97.2%) | 282 (96.9%) |  |  |
| Polypharmacy (*n*, %) | Yes | 303 (84.6%) | 242 (83.2%) | 0.667 | 0.04 |
|  | No | 55 (15.4%) | 49 (16.8%) |  |  |
| Oral hypoglycemic agent use (*n*, %) | Yes | 196 (54.7%) | 147 (50.5%) | 0.304 | 0.08 |
|  | No | 162 (45.3%) | 144 (49.5%) |  |  |
| Insulin regimen (*n*, %) | CSII | 11 (3.1%) | 54 (18.6%) | < 0.001 | 0.50 |
|  | MDI | 265 (74.0%) | 216 (74.2%) |  |  |
|  | LFIR | 82 (22.9%) | 21 (7.2%) |  |  |
| Insulin pump use (*n*, %) | Yes | 12 (3.4%) | 54 (18.6%) | < 0.001 | 0.49 |
|  | No | 346 (96.6%) | 237 (81.4%) |  |  |
| Total daily insulin dose [M (P_25_, P_75_), U] | | 31.2 (24.0, 41.2) | 30.0 (23.0, 39.0) | 0.365 | 0.08 |
| Premeal insulin use [M (P_25_, P_75_), U] | | 20.0 (15.0, 27.0) | 18.0 (12.0, 24.0) | 0.069 | 0.09 |
| Basal insulin use [M (P_25_, P_75_), U] | | 12.0 (8.0, 17.0) | 12.0 (8.0, 16.0) | 0.739 | 0.04 |
| Insulin type (*n*, %) | 1 | 96 (26.8%) | 79 (27.1%) | 0.412 | 0.09 |
|  | 2 | 257 (71.8%) | 203 (69.8%) |  |  |
|  | 3 | 5 (1.4%) | 8 (2.7%) |  |  |
|  | 4 | 0 (0.0%) | 1 (0.3%) |  |  |
| PPGE [M (P_25_, P_75_), mmol/L] | | 0.9 (-0.5, 2.3) | 0.3 (-1.1, 1.9) | 0.008 | 0.10 |
| LAGE [M (P_25_, P_75_), mmol/L] | | 6.1 (4.1, 8.6) | 8.7 (5.9, 12.1) | < 0.001 | 0.62 |
| Mean blood glucose [M (P_25_, P_75_), mmol/L] | | 9.3 (7.9, 10.9) | 9.7 (8.1, 11.4) | 0.220 | 0.05 |
| HbA1c [M (P_25_, P_75_), %] | | 10.8 (8.8, 13.3) | 10.2 (8.9, 12.5) | 0.062 | 0.09 |
| Fasting C-peptide [M (P_25_, P_75_), ng/mL] | | 0.7 (0.3, 1.3) | 0.2 (0.0, 0.6) | < 0.001 | 0.53 |
| 0.5-h C-peptide [M (P_25_, P_75_), ng/mL] | | 0.8 (0.4, 1.7) | 0.4 (0.1, 0.7) | < 0.001 | 0.59 |
| 1-h C-peptide [M (P_25_, P_75_), ng/mL] | | 1.1 (0.4, 2.2) | 0.4 (0.2, 1.0) | < 0.001 | 0.58 |
| 2-h C-peptide [M (P_25_, P_75_), ng/mL] | | 1.6 (0.7, 3.2) | 0.7 (0.2, 1.6) | < 0.001 | 0.03 |
| Serum creatinine [M (P_25_, P_75_), μmol/L] | | 61.2 (51.0, 75.0) | 62.8 (53.0, 75.0) | 0.274 | 0.14 |
| eGFR [M (P_25_, P_75_), mL/(min·1.73m²)] | | 107.2 (93.9, 120.2) | 103.8 (91.2, 113.2) | 0.001 | 0.27 |
| Total protein [M (P_25_, P_75_), g/L] | | 66.2 (60.8, 70.7) | 65.7 (58.8, 70.9) | 0.200 | 0.20 |
| Total cholesterol [M (P_25_, P_75_), mmol/L] | | 4.5 (3.8, 5.5) | 4.3 (3.7, 5.0) | 0.014 | 0.21 |
| Triglycerides [M (P_25_, P_75_), mmol/L] | | 1.3 (0.8, 1.8) | 1.0 (0.8, 1.5) | < 0.001 | 0.16 |
| LDL-C [M (P_25_, P_75_), mmol/L] | | 2.6 (2.0, 3.3) | 2.3 (1.9, 2.8) | < 0.001 | 0.32 |
| HDL-C [M (P_25_, P_75_), mmol/L] | | 1.2 (1.0, 1.4) | 1.4 (1.1, 1.7) | < 0.001 | 0.15 |
| Hemoglobin [M (P_25_, P_75_), g/L] | | 142.0 (130.0, 154.0) | 135.0 (125.0, 151.0) | < 0.001 | 0.28 |
| RBC count [M (P_25_, P_75_), ×10¹²/L] | | 4.7 (4.3, 5.2) | 4.6 (4.2, 4.9) | < 0.001 | 0.15 |
| WBC count [M (P_25_, P_75_), ×10^9^/L] | | 6.2 (5.2, 7.6) | 6.1 (4.9, 7.5) | 0.061 | 0.15 |
| CRP [M (P_25_, P_75_), mg/L] | | 3.1 (1.8, 4.8) | 3.6 (1.8, 6.3) | 0.024 | 0.20 |
| 25-hydroxyvitamin D [M (P_25_, P_75_), ng/mL] | | 29.8 (20.1, 41.3) | 27.1 (18.1, 38.0) | 0.074 | 0.12 |
| Urinary microalbumin [M (P_25_, P_75_), mg/L] | | 13.7 (8.7, 28.3) | 17.3 (10.5, 35.1) | 0.006 | 0.12 |
| Urinary protein [M (P_25_, P_75_)] | | 0.0 (0.0, 0.2) | 0.0 (0.0, 0.2) | 0.084 | 0.08 |
| GAD antibody (*n*, %) | Positive | 259 (72.3%) | 200 (68.7%) | 0.340 | 0.08 |
|  | Negative | 99 (27.7%) | 91 (31.3%) |  |  |
| ZnT8 antibody (*n*, %) | Positive | 49 (13.7%) | 29 (10.0%) | 0.182 | 0.12 |
|  | Negative | 309 (86.3%) | 262 (90.0%) |  |  |
| IAA (*n*, %) | Positive | 55 (15.4%) | 50 (17.2%) | 0.592 | 0.05 |
|  | Negative | 303 (84.6%) | 241 (82.8%) |  |  |
| IA-2 antibody (*n*, %) | Positive | 31 (8.7%) | 25 (8.6%) | 1.000 | 0.00 |
|  | Negative | 327 (91.3%) | 266 (91.4%) |  |  |
| ICA (*n*, %) | Positive | 23 (6.4%) | 38 (13.1%) | 0.015 | 0.22 |
|  | Negative | 335 (93.6%) | 253 (86.9%) |  |  |
| Serum potassium [M (P_25_, P_75_), mmol/L] | | 4.0 (3.8, 4.2) | 4.1 (3.9, 4.3) | 0.079 | 0.13 |
| Serum sodium [M (P_25_, P_75_), mmol/L] | | 139.0 (136.0, 141.0) | 139.0 (136.0, 141.0) | 0.349 | 0.10 |
| Serum calcium [M (P_25_, P_75_), mmol/L] | | 2.3 (2.2, 2.4) | 2.2 (2.1, 2.3) | < 0.001 | 0.19 |
| Length of stay [M (P_25_, P_75_), day] | | 8.0 (7.0, 10.0) | 10.0 (8.0, 13.0) | < 0.001 | 0.51 |

Table note: UEBMI, Urban Employee Basic Medical Insurance; URRBMI, Urban, Rural Resident Basic Medical Insurance; CSII, Continuous Subcutaneous Insulin Infusion; MDI, Multiple Daily Injections; LFIR, Low Frequency Insulin Regimen; PPGE, Postprandial Plasma Glucose Excursion; LAGE, Largest Amplitude of Glycemic Excursion; BMI, Body Mass Index; HbA1c, Glycated Hemoglobin A1c; C-peptide, Connecting peptide; eGFR, estimated Glomerular Filtration Rate; TC, Total Cholesterol; TG, Triglycerides; LDL-C, Low-Density Lipoprotein Cholesterol; HDL-C, High-Density Lipoprotein Cholesterol; RBC, Red Blood Cell; WBC, White Blood Cell; CRP, C-Reactive Protein; 25(OH)D, 25-hydroxyvitamin D; GAD, Glutamic Acid Decarboxylase antibody; ZnT8, Zinc Transporter 8 antibody; IAA, Insulin Autoantibody; IA-2, Insulinoma-Associated Antigen 2 antibody; ICA, Islet Cell Antibody; SMD, Standardized Mean Difference; Cr, Creatinine; U, Insulin unit; NA, Not Applicable.

Supplementary Table S6 Baseline characteristics stratified by hypoglycemia status in the external validation cohort

| **Variable** | | **No hypoglycemia**  **(*n* = 47)** | **Hypoglycemia**  **(*n* = 56)** | ***P* value** | **SMD** |
| --- | --- | --- | --- | --- | --- |
| Sex (*n*, %) | Male | 24 (51.1%) | 30 (53.6%) | 0.845 | 0.05 |
|  | Female | 23 (48.9%) | 26 (46.4%) |  |  |
| Age [M (P_25_, P_75_), year] | | 47.0 (33.5, 62.0) | 51.0 (36.0, 54.0) | 0.900 | 0.01 |
| Marital status (*n*, %) | Married | 34 (72.3%) | 41 (73.2%) | 0.749 | 0.21 |
|  | Unmarried | 4 (8.5%) | 2 (3.6%) |  |  |
|  | Divorced | 8 (17.0%) | 12 (21.4%) |  |  |
|  | Widowed | 1 (2.1%) | 1 (1.8%) |  |  |
| Ethnicity (*n*, %) | Han | 38 (80.9%) | 49 (87.5%) | 0.834 | 0.19 |
|  | Bai | 6 (12.8%) | 4 (7.1%) |  |  |
|  | Yi | 2 (4.3%) | 2 (3.6%) |  |  |
|  | Other minorities | 1 (2.1%) | 1 (1.8%) |  |  |
| Education level (*n*, %) | Illiterate/Primary school education | 16 (34.0%) | 25 (44.6%) | 0.564 | 0.22 |
|  | Junior /Senior high school | 22 (46.8%) | 22 (39.3%) |  |  |
|  | University and above | 9 (19.1%) | 9 (16.1%) |  |  |
| Insurance status (*n*, %) | UEBMI | 15 (31.9%) | 24 (42.9%) | 0.479 | 0.23 |
|  | URRBMI | 30 (63.8%) | 31 (55.4%) |  |  |
|  | Self-payment | 2 (4.3%) | 1 (1.8%) |  |  |
| BMI [M (P_25_, P_75_), kg/m^2^] | | 20.9 (19.5, 22.3) | 20.8 (19.5, 22.7) | 0.761 | 0.19 |
| Smoking status (*n*, %) | Yes | 22 (46.8%) | 22 (39.3%) | 0.549 | 0.15 |
|  | No | 25 (53.2%) | 34 (60.7%) |  |  |
| Duration of smoking [M (P_25_, P_75_), year] | | 6.0 (0.0, 13.9) | 13.9 (3.0, 23.6) | 0.011 | 0.43 |
| Alcohol use (*n*, %) | Yes | 7 (14.9%) | 8 (14.3%) | 1.000 | 0.02 |
|  | No | 40 (85.1%) | 48 (85.7%) |  |  |
| Duration of alcohol use [M (P25, P75), year] | | 0.0 (0.0, 4.7) | 3.1 (0.6, 9.6) | 0.004 | 0.22 |
| Age at diabetes onset [M (P25, P75), year] | | 50.0 (37.5, 56.5) | 44.5 (34.0, 58.2) | 0.508 | 0.14 |
| Diabetes duration [M (P25, P75), year] | | 5.0 (0.2, 8.8) | 7.0 (5.4, 10.0) | 0.053 | 0.26 |
| Previous hypoglycemia (*n*, %) | Yes | 10 (21.3%) | 26 (46.4%) | 0.012 | 0.53 |
|  | No | 37 (78.7%) | 30 (53.6%) |  |  |
| Family history of diabetes (*n*, %) | Yes | 9 (19.1%) | 4 (7.1%) | 0.081 | 0.36 |
|  | No | 38 (80.9%) | 52 (92.9%) |  |  |
| History of hypertension (*n*, %) | Yes | 6 (12.8%) | 9 (16.1%) | 0.781 | 0.09 |
|  | No | 41 (87.2%) | 47 (83.9%) |  |  |
| Ketosis (*n*, %) | Yes | 23 (48.9%) | 27 (48.2%) | 1.000 | 0.01 |
|  | No | 24 (51.1%) | 29 (51.8%) |  |  |
| Diabetic ketoacidosis (*n*, %) | Yes | 9 (19.1%) | 7 (12.5%) | 0.419 | 0.18 |
|  | No | 38 (80.9%) | 49 (87.5%) |  |  |
| Diabetic retinopathy (*n*, %) | Yes | 1 (2.1%) | 2 (3.6%) | 1.000 | 0.09 |
|  | No | 46 (97.9%) | 54 (96.4%) |  |  |
| Diabetic kidney disease (*n*, %) | Yes | 4 (8.5%) | 4 (7.1%) | 1.000 | 0.05 |
|  | No | 43 (91.5%) | 52 (92.9%) |  |  |
| Diabetic neuropathy (*n*, %) | Yes | 27 (57.4%) | 38 (67.9%) | 0.310 | 0.22 |
|  | No | 20 (42.6%) | 18 (32.1%) |  |  |
| Diabetic vascular disease (*n*, %) | Yes | 5 (10.6%) | 4 (7.1%) | 0.729 | 0.12 |
|  | No | 42 (89.4%) | 52 (92.9%) |  |  |
| Diabetic foot (*n*, %) | Yes | 0 (0.0%) | 1 (1.8%) | 1.000 | 0.19 |
|  | No | 47 (100.0%) | 55 (98.2%) |  |  |
| Number of diabetic complications [M (P_25_, P_75_)] | | 1.0 (1.0, 2.0) | 1.0 (1.0, 2.0) | 0.617 | 0.03 |
| Sleep disorder (*n*, %) | Yes | 1 (2.1%) | 0 (0.0%) | 0.456 | 0.21 |
|  | No | 46 (97.9%) | 56 (100.0%) |  |  |
| Polypharmacy (*n*, %) | Yes | 34 (72.3%) | 39 (69.6%) | 0.830 | 0.06 |
|  | No | 13 (27.6%) | 17 (30.4%) |  |  |
| Oral hypoglycemic agent use (*n*, %) | Yes | 18 (38.3%) | 16 (28.6%) | 0.400 | 0.21 |
|  | No | 29 (61.7%) | 40 (71.4%) |  |  |
| Insulin regimen (*n*, %) | CSII | 3 (6.4%) | 14 (25.0%) | 0.002 | 0.72 |
|  | MDI | 40 (85.1%) | 29 (51.8%) |  |  |
|  | LFIR | 4 (8.5%) | 13 (23.2%) |  |  |
| Insulin pump use (*n*, %) | Yes | 2 (4.3%) | 6 (10.7%) | 0.285 | 0.25 |
|  | No | 45 (95.7%) | 50 (89.3%) |  |  |
| Total daily insulin dose [M (P_25_, P_75_), U] | | 31.0 (25.4, 36.6) | 29.5 (22.3, 35.3) | 0.448 | 0.15 |
| Premeal insulin use [M (P_25_, P_75_), U] | | 18.0 (14.7, 25.3) | 18.0 (11.7, 21.2) | 0.122 | 0.42 |
| Basal insulin use [M (P_25_, P_75_), U] | | 12.1 (7.0, 15.9) | 14.0 (10.0, 16.0) | 0.088 | 0.37 |
| Insulin type (*n*, %) | 1 | 5 (10.6%) | 30 (53.6%) | < 0.001 | 0.98 |
|  | 2 | 42 (89.4%) | 24 (42.9%) |  |  |
|  | 3 | 0 (0.0%) | 2 (3.6%) |  |  |
|  | 4 | 0 (0.0%) | 0 (0.0%) |  |  |
| PPGE [M (P_25_, P_75_), mmol/L] | | 1.2 (0.7, 2.6) | 2.2 (0.5, 3.7) | 0.080 | 0.35 |
| LAGE [M (P_25_, P_75_), mmol/L] | | 7.3 (5.3, 9.7) | 7.7 (6.0, 12.4) | 0.286 | 0.25 |
| Mean blood glucose [M (P_25_, P_75_), mmol/L] | | 9.2 (7.8, 11.2) | 9.6 (8.2, 11.7) | 0.472 | 0.06 |
| HbA1c [M (P_25_, P_75_), %] | | 10.3 (8.3, 12.3) | 9.6 (8.7, 10.5) | 0.460 | 0.22 |
| Fasting C-peptide [M (P_25_, P_75_), ng/mL] | | 0.2 (0.0, 0.6) | 0.0 (0.0, 0.1) | < 0.001 | 0.33 |
| 0.5-h C-peptide [M (P_25_, P_75_), ng/mL] | | 0.6 (0.1, 0.8) | 0.0 (0.0, 0.1) | < 0.001 | 0.81 |
| 1-h C-peptide [M (P_25_, P_75_), ng/mL] | | 0.7 (0.0, 0.8) | 0.0 (0.0, 0.2) | < 0.001 | 0.65 |
| 2-h C-peptide [M (P_25_, P_75_), ng/mL] | | 0.7 (0.0, 1.5) | 0.0 (0.0, 0.2) | < 0.001 | 0.70 |
| Serum creatinine [M (P_25_, P_75_), μmol/L] | | 56.1 (47.0, 68.5) | 59.1 (49.1, 70.2) | 0.551 | 0.17 |
| eGFR [M (P_25_, P_75_), mL/(min·1.73m²)] | | 106.8 (96.6, 123.5) | 106.4 (94.8, 123.3) | 0.971 | 0.01 |
| Total protein [M (P_25_, P_75_), g/L] | | 70.1 (66.3, 74.6) | 69.9 (64.1, 74.1) | 0.567 | 0.09 |
| Total cholesterol [M (P_25_, P_75_), mmol/L] | | 4.6 (4.0, 5.3) | 4.2 (3.7, 5.0) | 0.086 | 0.33 |
| Triglycerides [M (P_25_, P_75_), mmol/L] | | 1.1 (0.8, 1.5) | 1.1 (0.8, 1.2) | 0.231 | 0.22 |
| LDL-C [M (P_25_, P_75_), mmol/L] | | 2.5 (2.1, 3.0) | 2.3 (1.8, 2.8) | 0.049 | 0.32 |
| HDL-C [M (P_25_, P_75_), mmol/L] | | 1.3 (1.2, 1.6) | 1.4 (1.2, 1.6) | 0.708 | 0.07 |
| Hemoglobin [M (P_25_, P_75_), g/L] | | 142.5 (127.9, 154.8) | 137.5 (122.8, 151.0) | 0.292 | 0.20 |
| RBC count [M (P_25_, P_75_), ×10¹²/L] | | 4.9 (4.4, 5.6) | 4.8 (4.5, 5.5) | 0.733 | 0.13 |
| WBC count [M (P_25_, P_75_), ×10^9^/L] | | 6.5 (5.4, 8.4) | 7.0 (5.0, 8.7) | 0.884 | 0.02 |
| CRP [M (P_25_, P_75_), mg/L] | | 2.8 (0.4, 3.9) | 2.5 (0.9, 5.0) | 0.761 | 0.02 |
| 25-hydroxyvitamin D [M (P_25_, P_75_), ng/mL] | | 18.7 (14.3, 22.6) | 19.6 (14.2, 24.7) | 0.460 | 0.29 |
| Urinary microalbumin [M (P_25_, P_75_), mg/L] | | 11.1 (10.9, 17.9) | 17.0 (11.0, 41.3) | 0.004 | 0.45 |
| Urinary protein [M (P_25_, P_75_)] | | 0.0 (0.0, 0.0) | 0.0 (0.0, 0.0) | 0.428 | 0.16 |
| GAD antibody (*n*, %) | Positive | 41 (87.2%) | 24 (42.9%) | < 0.001 | 0.93 |
|  | Negative | 6 (12.8%) | 32 (57.1%) |  |  |
| ZnT8 antibody (*n*, %) | Positive | 16 (34.0%) | 16 (28.6%) | 0.670 | 0.12 |
|  | Negative | 31 (66.0%) | 40 (71.4%) |  |  |
| IAA (*n*, %) | Positive | 8 (17.0%) | 18 (32.1%) | 0.111 | 0.35 |
|  | Negative | 39 (83.0%) | 38 (67.9%) |  |  |
| IA-2 antibody (*n*, %) | Positive | 8 (17.0%) | 6 (10.7%) | 0.397 | 0.18 |
|  | Negative | 39 (83.0%) | 50 (89.3%) |  |  |
| ICA (*n*, %) | Positive | 15 (31.9%) | 15 (26.8%) | 0.665 | 0.11 |
|  | Negative | 32 (68.1%) | 41 (73.2%) |  |  |
| Serum potassium [M (P_25_, P_75_), mmol/L] | | 4.1 (3.9, 4.3) | 4.1 (3.9, 4.4) | 0.892 | 0.16 |
| Serum sodium [M (P_25_, P_75_), mmol/L] | | 138.8 (136.2, 139.9) | 139.0 (137.5, 140.2) | 0.529 | 0.17 |
| Serum calcium [M (P_25_, P_75_), mmol/L] | | 2.3 (2.2, 2.4) | 2.3 (2.2, 2.4) | 0.952 | 0.15 |
| Length of stay [M (P_25_, P_75_), day] | | 8.0 (6.0, 10.5) | 9.0 (7.0, 13.0) | 0.270 | 0.35 |

Table note: UEBMI, Urban Employee Basic Medical Insurance; URRBMI, Urban, Rural Resident Basic Medical Insurance; CSII, Continuous Subcutaneous Insulin Infusion; MDI, Multiple Daily Injections; LFIR, Low Frequency Insulin Regimen; PPGE, Postprandial Plasma Glucose Excursion; LAGE, Largest Amplitude of Glycemic Excursion; BMI, Body Mass Index; HbA1c, Glycated Hemoglobin A1c; C-peptide, Connecting peptide; eGFR, estimated Glomerular Filtration Rate; TC, Total Cholesterol; TG, Triglycerides; LDL-C, Low-Density Lipoprotein Cholesterol; HDL-C, High-Density Lipoprotein Cholesterol; RBC, Red Blood Cell; WBC, White Blood Cell; CRP, C-Reactive Protein; 25(OH)D, 25-hydroxyvitamin D; GAD, Glutamic Acid Decarboxylase antibody; ZnT8, Zinc Transporter 8 antibody; IAA, Insulin Autoantibody; IA-2, Insulinoma-Associated Antigen 2 antibody; ICA, Islet Cell Antibody; SMD, Standardized Mean Difference; Cr, Creatinine; U, Insulin unit; NA, Not Applicable.

Supplementary Table S7 Final model hyperparameters

| **Model** | **Hyperparameter** | **Final value** |
| --- | --- | --- |
| Logistic regression | C | 35.5182 |
| Random forest | n_estimators | 100 |
|  | max_depth | 8 |
|  | min_samples_split | 3 |
|  | min_samples_leaf | 14 |
|  | max_features | 0.3226 |
| XGBoost | n_estimators | 450 |
|  | max_depth | 6 |
|  | learning_rate | 0.0359 |
|  | subsample | 0.6311 |
|  | colsample_bytree | 0.8854 |
|  | gamma | 4.8114 |
|  | min_child_weight | 4 |
|  | reg_alpha | 0.3786 |
|  | reg_lambda | 1.5382 |
|  | max_delta_step | 2 |

Table note: Final hyperparameters were selected by Optuna in the training set. Only model-specific tuned hyperparameters are shown.

Supplementary Table S8 Pairwise DeLong tests for AUROC comparisons among prediction models

| **Dataset** | **Model comparison** | | ***n*** | **Events** | **AUROC of model 1** | **AUROC of model 2** | **AUROC difference** | ***P* value** |
| --- | --- | --- | --- | --- | --- | --- | --- | --- |
|  | **Model 1** | **Model 2** |  |  |  |  |  |  |
| Internal validation | Logistic regression | Random forest | 194 | 87 | 0.810 | 0.791 | 0.019 | 0.037 |
| Internal validation | Logistic regression | XGBoost | 194 | 87 | 0.810 | 0.811 | -0.001 | 0.577 |
| Internal validation | Random forest | XGBoost | 194 | 87 | 0.791 | 0.811 | -0.020 | 0.010 |
| External validation | Logistic regression | Random forest | 103 | 56 | 0.753 | 0.735 | 0.018 | 0.497 |
| External validation | Logistic regression | XGBoost | 103 | 56 | 0.753 | 0.770 | -0.017 | 0.562 |
| External validation | Random forest | XGBoost | 103 | 56 | 0.735 | 0.770 | -0.035 | 0.116 |

Table note: Pairwise AUROC comparisons were conducted using the DeLong tests based on paired model predictions within the same validation cohort. The AUROC difference was calculated as the AUROC of model 1 minus that of model 2. A P values < 0.05 was defined as statistically significant. Abbreviations. AUROC, area under the receiver operating characteristic curve; LR, logistic regression; RF, random forest; XGBoost, extreme gradient boosting.

Supplementary Table S9 Sensitivity analyses for the final XGBoost model after excluding individual predictors

| Predictor excluded | External AUROC | AUPRC | Brier score | F1 score |
| --- | --- | --- | --- | --- |
| None | 0.770 | 0.773 | 0.202 | 0.777 |
| LAGE | 0.763 | 0.746 | 0.203 | 0.780 |
| Fasting C-peptide | 0.672 | 0.716 | 0.223 | 0.724 |
| HbA1c | 0.764 | 0.768 | 0.206 | 0.755 |
| Sex | 0.756 | 0.758 | 0.206 | 0.777 |
| Insulin pump use | 0.748 | 0.762 | 0.211 | 0.768 |
| Previous hypoglycemia | 0.742 | 0.745 | 0.208 | 0.777 |

Table note: Sensitivity analyses were performed by refitting the final XGBoost model after removing one predictor at a time. Model performance was evaluated using external AUROC, AUPRC, Brier score, and F1 score.

Supplementary Table S10 Missing-data sensitivity analyses for the final XGBoost model

| **Missing-data strategy** | **External AUROC** | **AUPRC** | **Brier score** | **F1 score** |
| --- | --- | --- | --- | --- |
| Median imputation | 0.758 | 0.761 | 0.208 | 0.764 |
| Missing-indicator approach | 0.751 | 0.754 | 0.211 | 0.758 |
| Complete-case analysis | 0.742 | 0.739 | 0.216 | 0.746 |
| KNN imputation | 0.766 | 0.772 | 0.204 | 0.773 |

Table note: The primary analysis adopted multiple imputation by chained equations (MICE). Alternative strategies included median imputation, missing-indicator method, complete-case analysis and KNN imputation.

Supplementary Table S11 Sensitivity analysis in antibody-complete subsets with and without islet autoantibody markers for the final XGBoost model

| **Dataset** | **Predictors** | **Sample size (*n*)** | **Hypoglycemia events (*n*)** | **AUROC** | **AUPRC** | **Brier score** |
| --- | --- | --- | --- | --- | --- | --- |
| Internal antibody-complete subset | Original predictors | 54 | 27 | 0.781 | 0.769 | 0.202 |
| External antibody-complete subset | Original predictors | 56 | 24 | 0.711 | 0.615 | 0.237 |
| Internal antibody-complete subset | Original + GAD/IAA/ZnT8/IA-2/ICA | 54 | 27 | 0.770 | 0.763 | 0.202 |
| External antibody-complete subset | Original + GAD/IAA/ZnT8/IA-2/ICA | 56 | 24 | 0.710 | 0.615 | 0.237 |

Table note: Analyses were restricted to participants with complete islet autoantibody data. The expanded model integrated five autoantibody markers based on the original six predictors.

Supplementary Table S12 Subgroup performance of the final model in the internal validation set

| **Variable** | **Subgroup** | **Sample size (*n*)** | **Hypoglycemia events (*n*)** | **Event rate (%)** | **Sensitivity** | **Specificity** | **PPV** | **NPV** |
| --- | --- | --- | --- | --- | --- | --- | --- | --- |
| Sex | Female | 66 | 32 | 48.5 | 0.875 | 0.647 | 0.700 | 0.846 |
|  | Male | 128 | 55 | 43.0 | 0.636 | 0.699 | 0.614 | 0.718 |
| Age | < 40 years | 57 | 18 | 31.6 | 0.667 | 0.667 | 0.480 | 0.812 |
|  | 40–60 years | 87 | 48 | 55.2 | 0.688 | 0.718 | 0.750 | 0.651 |
|  | > 60 years | 50 | 21 | 42.0 | 0.857 | 0.655 | 0.643 | 0.864 |
| Ethnicity | Ethnicity 1 | 138 | 52 | 37.7 | 0.731 | 0.674 | 0.576 | 0.806 |
|  | Ethnicity 2 | 32 | 20 | 62.5 | 0.750 | 0.667 | 0.789 | 0.615 |
|  | Ethnicity 3+4 | 24 | 15 | 62.5 | 0.667 | 0.778 | 0.833 | 0.583 |
| Insulin pump use | No pump | 174 | 69 | 39.7 | 0.652 | 0.695 | 0.584 | 0.753 |
|  | With pump | 20 | 18 | 90.0 | 1.000 | 0.000 | 0.900 | NA |
| Previous hypoglycemia | No history | 157 | 65 | 41.4 | 0.677 | 0.728 | 0.638 | 0.761 |
|  | With history | 37 | 22 | 59.5 | 0.864 | 0.400 | 0.679 | 0.667 |
| HbA1c | ≥ 7.5% | 169 | 77 | 45.6 | 0.727 | 0.663 | 0.644 | 0.753 |
| LAGE | 2.5–5 | 47 | 14 | 29.8 | 0.643 | 0.909 | 0.750 | 0.857 |
|  | ≥ 5 | 138 | 69 | 50.0 | 0.754 | 0.565 | 0.634 | 0.696 |
| Fasting C-peptide | < 0.3 | 64 | 41 | 64.1 | 0.927 | 0.217 | 0.679 | 0.625 |
|  | 0.3–0.7 | 70 | 31 | 44.3 | 0.613 | 0.615 | 0.559 | 0.667 |
|  | > 0.7 | 60 | 15 | 25.0 | 0.400 | 0.978 | 0.857 | 0.824 |

Table note: Subgroup analyses were performed in the internal validation set. Given small sample sizes and limited events in certain strata, all subgroup results should be interpreted cautiously. NA indicates that negative predictive value could not be reliably estimated. For HbA1c and LAGE subgroup analyses, only clinically prespecified strata with sufficient sample size are shown.
